# Supplementary material for: Chemical genetics reveals Leishmania KKT2 and CRK9 kinase activity is required for cell cycle progression
Source: PLoS Pathog. 2026 May 13;22(5):e1014194. doi: 10.1371/journal.ppat.1014194 (PMC13211308; doi:10.1371/journal.ppat.1014194)
Supplement: S1 Table — (PDF) [file ppat.1014194.s001.pdf]

S1 Table – Protein kinase genes previously identified by our group as required in *L. mexicana* promastigotes.

Gene ID	Group/Family	Name	Reference
LmxM.03.0780	AGC/RSK		Baker et al., 2021 [1]
LmxM.25.2340	AGC	AEK1	Baker et al., 2021 [1]
LmxM.29.1000	AGC		Baker et al., 2021 [1]
LmxM.34.4010	AGC/PKA	PKAC1	Baker et al., 2021 [1]
LmxM.04.0650	CAMK/CAMKL		Baker et al., 2021 [1]
LmxM.27.1780	CK1/CK1	CK1.4	Baker et al., 2021 [1]
LmxM.34.1010	CK1/CK1	CK1.2	Baker et al., 2021 [1]
LmxM.05.0550	CMGC/CDK	CRK2	Baker et al., 2021 [1]
LmxM.09.0310	CMGC/CDK	CRK12	Baker et al., 2021 [1]
LmxM.14.0830	CMGC/DYRK		Baker et al., 2021 [1]
LmxM.15.0180	CMGC/DYRK	DYRK1	Baker et al., 2021 [1]
LmxM.18.0270	CMGC/GSK	GSK3	Baker et al., 2021 [1]
LmxM.19.1440	CMGC/MAPK	MPK4	Baker et al., 2021 [1]
LmxM.21.1080	CMGC/CDK	CRK1	Baker et al., 2021 [1]
LmxM.22.0490	CMGC/MAPK	GSK3a	Baker et al., 2021 [1]
LmxM.24.0670	CMGC	MSK	Baker et al., 2021 [1]
LmxM.27.1940	CMGC/CDK	CRK9	Baker et al., 2021 [1]
LmxM.29.1780	CMGC/CDK	CRK11	Baker et al., 2021 [1]
LmxM.36.0550	CMGC/CDK	CRK3	Baker et al., 2021 [1]
LmxM.08_29.1330	Other/AUR	AUK2	Baker et al., 2021 [1]
LmxM.08.0530	Other/unique		Baker et al., 2021 [1]
LmxM.17.0790	Other/PLK	PLK	Baker et al., 2021 [1]
LmxM.20.0960	Other/unique		Baker et al., 2021 [1]
LmxM.20.1330	Other/unique		Baker et al., 2021 [1]
LmxM.26.2110	Other/Orphan		Baker et al., 2021 [1]
LmxM.28.0520	Other/AUR	AUK1/AIRK	Baker et al., 2021 [1]
LmxM.28.1760	Other/VPS15		Baker et al., 2021 [1]
LmxM.30.2860	Other/TLK	TLK	Baker et al., 2021 [1]
LmxM.34.4050	Other/Orphan	KKT3	Baker et al., 2021 [1]
LmxM.36.5350	Other/Orphan	KKT2	Baker et al., 2021 [1]
LmxM.02.0290	NEK		Baker et al., 2021 [1]
LmxM.30.2960	NEK	RDK2	Baker et al., 2021 [1]
LmxM.07.0690	STE		Baker et al., 2021 [1]
LmxM.14.1300	STE		Baker et al., 2021 [1]
LmxM.17.0490	STE		Baker et al., 2021 [1]
LmxM.24.2320	STE	MKK4**	Baker et al., 2021 [1]
LmxM.25.1990	STE		Baker et al., 2021 [1]
LmxM.27.1370	STE		Baker et al., 2021 [1]
LmxM.34.3170	STE		Baker et al., 2021 [1]
LmxM.36.0860	STE		Baker et al., 2021 [1]
LmxM.24.2010	Atypical	PI3K	Baker et al., 2021 [1]
LmxM.33.4530	Atypical	TOR2	Baker et al., 2021 [1]
LmxM.36.6320	Atypical	TOR1	Baker et al., 2021 [1]
LmxM.09.0400 *	CMGC/CLK	CLK1/KKT10	Geoghegan et al., 2022 [2]; Catta-Preta et al., 2025[3]
LmxM.09.0410 *	CMGC/CLK	CLK2/KKT19	Geoghegan et al., 2022 [2]; Catta-Preta et al., 2025[3]

* Protein kinases that exhibit biological redundancy were classified as “required” for parasite survival, since simultaneous inhibition of the redundant pair resulted in loss of viability.
** Protein kinase successfully deleted from the *L. mexicana* genome using homologous recombination [4].

References

1. Baker N, Catta-Preta CMC, Neish R, Sadlova J, Powell B, Alves-Ferreira EVC, et al. Systematic functional analysis of Leishmania protein kinases identifies regulators of differentiation or survival. Nat Commun. 2021;12(1):1244. Epub 2021/02/25. doi: 10.1038/s41467-021-21360-8. PubMed PMID: 33623024; PubMed Central PMCID: PMCPMC7902614.

2. Geoghegan V, Carnielli JBT, Jones NG, Saldivia M, Antoniou S, Hughes C, et al. CLK1/CLK2-driven signalling at the Leishmania kinetochore is captured by spatially referenced proximity phosphoproteomics. Commun Biol. 2022;5(1):1305. Epub 20221128. doi: 10.1038/s42003-022-04280-1. PubMed PMID: 36437406; PubMed Central PMCID: PMCPMC9701682.

3. Catta-Preta CMC, Ramos PZ, Carnielli JBT, Vasconcelos SNS, Dowle A, Fanti RC, et al. Discovery and Characterization of Cell-Permeable Inhibitors of Leishmania mexicana CLK1 Using an In-Cell Target Engagement Assay. ACS Infect Dis. 2025;11(10):2859-70. Epub 20250925. doi: 10.1021/acsinfecdis.5c00610. PubMed PMID: 40994280.

4. Kuhn D, Wiese M. LmxPK4, a mitogen-activated protein kinase kinase homologue of Leishmania mexicana with a potential role in parasite differentiation. Mol Microbiol. 2005;56(5):1169-82. Epub 2005/05/11. doi: 10.1111/j.1365-2958.2005.04614.x. PubMed PMID: 15882412.
