# Supplementary material for: Chemical genetics reveals Leishmania KKT2 and CRK9 kinase activity is required for cell cycle progression
Source: PLoS Pathog. 2026 May 13;22(5):e1014194. doi: 10.1371/journal.ppat.1014194 (PMC13211308; doi:10.1371/journal.ppat.1014194)
Supplement: S2 Table — (PDF) [file ppat.1014194.s002.pdf]

S2 Table – Sequence of oligonucleotides used to generate sgRNA and DNA repair template for CRISPR-Cas9 edited lines.

Oligo ID	Engineered cell line	Sequence (5' → 3')	Description
OL6137 (R)	N/A	AAAAGCACCGACTCGGTGCCACTTTTTCAAGTTGATAACGGACTAGCCTTATTTTAACTTGCTATTTCTAGCTCTAAAAC	Reverse oligo to generate 5'sgRNA and 3'sgRNA
OL11240 (F)	AS CLK1^{M213} / AS CLK2^{M220}	GAAATTAATACGACTCACTATAGGCGCTATTTCCAGAACGACAGGTTTTAGAGCTAGAAATAGC	5'sgRNA
OL11241 (F)		GAAATTAATACGACTCACTATAGGCAAGTACGGCCCCTGCCTGCGTTTTAGAGCTAGAAATAGC	3'sgRNA
OL11613 (F)	AS KKT2^{M146}	GAAATTAATACGACTCACTATAGGCAAATTCTACGGTGCTGGTAGTTTTAGAGCTAGAAATAGC	5'sgRNA
OL11614 (F)		GAAATTAATACGACTCACTATAGGGTAGTAATGGAGCGGTGCGCGTTTTAGAGCTAGAAATAGC	3'sgRNA
OL11607 (F)	AS KKT3^{M110}	GAAATTAATACGACTCACTATAGGACAGCGGACTTGATCGTTATGTTTTAGAGCTAGAAATAGC	5'sgRNA
OL11608 (F)		GAAATTAATACGACTCACTATAGGAGAAGGTCGTGGAGCGTGCTGTTTTAGAGCTAGAAATAGC	3'sgRNA
OL11601 (F)	AS CRK9^{M501}	GAAATTAATACGACTCACTATAGGGAAGGACGTCTTCCTTGTAAGTTTTAGAGCTAGAAATAGC	5'sgRNA
OL11602 (F)		GAAATTAATACGACTCACTATAGGACTACTGTCCCTACGACCTGGTTTTAGAGCTAGAAATAGC	3'sgRNA
OL14683 (F)	Δ*kkt3*	GAAATTAATACGACTCACTATAGGCTATGTGTGGTGGGCGATTTGTTTTAGAGCTAGAAATAGC	5'sgRNA
OL14685 (F)		GAAATTAATACGACTCACTATAGGTTCCCGTCGTTTTCGCTCACGTTTTAGAGCTAGAAATAGC	3'sgRNA
OL14681 (F)		AGATTTTGTTGTCTATCACAATCAAAGTAGGTGTATCGGATGTCAGTTGCGTGGCTGATGTCCGTATGTATAATGCAGACCTGCTGC	Upstream forward to generate repair template
OL14684 (R)		CTTTTTTTTTCCGCATCCACGACCTCTGCACCAATTTGAGAGACCTGTGC	Downstream reverse to generate repair template
OL6945 (F)	KKT2::mNG::3xMyc	GAAATTAATACGACTCACTATAGGGAGGACAGCGCGTCGTGAGTGTTTTAGAGCTAGAAATAGC	3'sgRNA
OL6943 (F)	KKT2_AS::mNG::3xMyc	GAGACGGTCCTCAACAACAATTTCCGGAGAGGTTCTGGTAGTGGTTCCGG	Downstream forward to generate repair template
OL6944 (R)	KKT2_AS::3xMyc::mT	AGAAGATACACGTAAACACGAACTCAACCACCAATTTGAGAGACCTGTGC	Downstream reverse to generate repair template
OL15542 (R)	KKT3$^{D157A-D174A}$ or KKT3$^{D157D-D174D}$	GAAATTAATACGACTCACTATAGGACGAGACGGCTTGAACCGCCGTTTTAGAGCTAGAAATAGC	5'sgRNA
OL15543 (F)		GAAATTAATACGACTCACTATAGGTTGTAACTCGCTTAAGCCACGTTTTAGAGCTAGAAATAGC	3'sgRNA
OL15538 (F)		CGTATCACCAGCGAAGCGGAGATGCTTTTCATGGCACATCAAGCCGTACAGGCGGTGTCGTACGTGCACGCAGAGGGCTGCATTCACCGCGCGATCAAGCTTCAGAACTTTGTCTTCGAC	Forward primer to generate KKT3$^{D157A-D174A}$ repair template
OL15539 (R)		AGACATGAAAGACACAGTGCCCGCCACCACGTCGCCTGCTGGAGGTTTTAGGGAATTGCAAGCAAGGCCAAACGCGATCAGCTTCAGATTACCATCGAGGTCGAAGACAAAGTTCTGAA	Reverse primer to generate KKT3$^{D157A-D174A}$ repair template
OL15540 (F)		CGTATCACCAGCGAAGCGGAGATGCTTTTCATGGCACATCAAGCCGTACAGGCGGTGTCGTACGTGCACGCAGAGGGCTGCATTCACCGCGATATCAAGCTTCAGAACTTTGTCTTCGAC	Forward primer to generate KKT3$^{D157D-D174D}$ repair template
OL15541 (R)		AGACATGAAAGACACAGTGCCCGCCACCACGTCGCCTGCTGGAGGTTTTAGGGAATTGCAAGCAAGGCCAAAATCGATCAGCTTCAGATTACCATCGAGGTCGAAGACAAAGTTCTGAA	Reverse primer to generate KKT3$^{D157D-D174D}$ repair template
OL15548 (F)	KKT3^{K64A} or KKT3^{K64K}	GAAATTAATACGACTCACTATAGGCTGGCCCTCAAAATTTCAACGTTTTAGAGCTAGAAATAGC	5'sgRNA
OL15544 (F)		CGCATTGGCCAAGGCTCCTTCGGCACGGTGTACCGCGCCGTCAGCAGCGACTATCCACGTCTCGCGCTGGCTATCTCTAC	Forward primer to generate KKT3^{K64A} repair template
OL15545 (R)		CCGTCCTTTCGTACACACACGACTCAGCACATCTAACTCCTGGCGAAGCCGCGTACTCTTGCCTGTAGAGATAGCCAGCGCGAG	Reverse primer to generate KKT3^{K64A} repair template
OL15546 (F)		CGCATTGGCCAAGGCTCCTTCGGCACGGTGTACCGCGCCGTCAGCAGCGACTATCCACGTCTCGCGCTGAAGATCTCTAC	Forward primer to generate KKT3^{K64K} repair template
OL15547 (R)		CCGTCCTTTCGTACACACACGACTCAGCACATCTAACTCCTGGCGAAGCCGCGTACTCTTGCCTGTAGAGATCTTCAGCGCGAG	Reverse primer to generate KKT3^{K64K} repair template

Oligo ID	Engineered cell line	Sequence (5' → 3')	Description
OL15551 (R)	KKT3^{43_327del}	GAAATTAATACGACTCACTATAGGACACCGTGCCGAAGGAGCCTGTTTTAGAGCTAGAAATAGC	5'sgRNA
OL15552 (R)		GAAATTAATACGACTCACTATAGGGCTCACACTCGGCCGCCCCTGTTTTAGAGCTAGAAATAGC	3'sgRNA
OL15549 (F)		CGTAGCGGCGATTCGGAGATCGTGAAGGAGGTGTGGCCGTGCGAGCGCATTGGTTCTGGTAGTGGTTCCGGTTCC	Forward primer to generate KKT3^{43_327del} repair template
OL15550 (R)		TGCAGGTGGATAGCGCCGCCGTAAGTTCCACAGCTTGTTCTCCAGGATTGTAGAACCGGAACCGGAACCACTACCAGAACCA GAACCGGAACCGGAACCACTACCAG	Reverse primer to generate KKT3^{43_327del} repair template

R, reverse oligo; F, forward oligo; N/A, not applicable; AS, analog-sensitive kinase; *Δ*, knockout target gene; del, deletion.

The R sgRNA oligo OL6137 holds the Cas9 scaffold (black) and the complementary sequence to the forward primer (blue). The F oligo sequences to generate sgRNA are coloured as follows: T7 promoter in black; sgRNA target sites in red; complementary sequence to sgRNA scaffold in blue. To generate linear DNA fragment for *in vivo* transcription of the single guide RNA, Q5 High-Fidelity DNA Polymerase (New England BioLabs inc. Cat. M0491L) was used in a 40 µL reaction mix containing 1x Q5 Reaction buffer, 200 µM dNTPs, 2 µM of the sgRNA scaffold oligo (OL6137), 2 µM of the forward oligo and 0.8 units of Q5 High-Fidelity DNA Polymerase. Cycling conditions: initial denaturation at 98ºC for 30 seconds, followed by 40 cycles of 98ºC for 10 seconds (denaturation), 60ºC for 30 seconds (annealing), 72ºC for 15 seconds (extension) and a final extension step at 72ºC for 10 minutes.

The upstream and downstream oligo sequences to generate DNA repair template for gene deletion are coloured as follows: 30 bp for homology direct recombination in red; barcode in green; complementary sequence to the template plasmid in blue. DNA repair template was generated using Q5 High-Fidelity DNA Polymerase in a 40 µL reaction mix containing 1x Q5 Reaction buffer, 200 µM dNTPs, 2 µM of each forward and reverse oligos, 0.6 ng of the template and 0.8 units of Q5 High-Fidelity DNA Polymerase. Cycling conditions: initial denaturation at 94ºC for 5 minutes followed by 45 cycles of 94ºC for 30 seconds (denaturation), 65ºC for 30 seconds (annealing), 72ºC for 2.25 minutes (extension) and a final extension step at 72ºC for 10 minutes. Templates used to generate DNA repair template to knockout and endogenously tag genes (sequences available on http://leishgedit.net/): pPLOTv1 blast-mNeonGreen-blast; pPLOTv1 puro-mNeonGreen-puro; pTBlast_v1; pTPuro_v1.

DNA repair template for drug-free CRISPR-Cas9 was generated using Q5 High-Fidelity DNA Polymerase in a 5x 40 µL reaction mix containing 1x Q5 Reaction buffer, 200 µM dNTPs, 2 µM of each forward and reverse oligos, and 0.8 units of Q5 High-Fidelity DNA Polymerase. Cycling conditions: initial denaturation at 94ºC for 5 minutes followed by 35 cycles of 98ºC for 30 seconds (denaturation), 60ºC for 30 seconds (annealing), 72ºC for 20 seconds (extension) and a final extension step at 72ºC for 10 minutes.
