# Supplementary material for: Chemical genetics reveals Leishmania KKT2 and CRK9 kinase activity is required for cell cycle progression
Source: PLoS Pathog. 2026 May 13;22(5):e1014194. doi: 10.1371/journal.ppat.1014194 (PMC13211308; doi:10.1371/journal.ppat.1014194)
Supplement: S3 Table — (PDF) [file ppat.1014194.s003.pdf]

S3 Table – Sequence of the DNA repair templates used to engineer analog-sensitive kinases in *Leishmania*.

Engineered cell line	Sequence
AS CLK1^{M213G} / AS CLK2^{M220G}	GACCGCTTCCCGCTGATGAAGATCCAGCGTTACTTTCAAAATGATTCTGGTCATATGTGCATCGTC**GGC**CCCAAATATGGACCGTGTCTCCTAGACTGGATCATGAAGCACGGCCCCTTC (ssDRT)
AS CLK1^{M213A} / AS CLK2^{M220A}	GACCGCTTCCCGCTGATGAAGATCCAGCGTTACTTTCAAAATGATTCTGGTCATATGTGCATCGTC**GCG**CCCAAATATGGACCGTGTCTCCTAGACTGGATCATGAAGCACGGCCCCTTC (ssDRT)
AS KKT2^{M146G}	GAGCCATTTCTCGCGCCATCCCAACATTGTCAAGTTTTATGGAGCGGGCCGCGACGAGGACCGAGCGTATGTGGTG**GGC**GAACGTTGTGCAGGCAAGTCGCTTCACGACGTCATAGCCAG (ssDRT)
AS KKT2^{M146A}	GAGCCATTTCTCGCGCCATCCCAACATTGTCAAGTTTTATGGAGCGGGCCGCGACGAGGACCGAGCGTATGTGGTG**GCG**GAACGTTGTGCAGGCAAGTCGCTTCACGACGTCATAGCCAG (ssDRT)
AS KKT3^{M110G}	CGCGTTTCGAGTTTGGAGCGCTCAACAAGACGGCAGATCTCATTGTGATCGGA**GGC**GAACTATGCGTCCCCAGTACTCTGCATGATTTGCTCCTCAGCACTCGTATCACCAGCGAAGCGG (ssDRT)
AS KKT3^{M110A}	CGCGTTTCGAGTTTGGAGCGCTCAACAAGACGGCAGATCTCATTGTGATCGGA**GCG**GAACTATGCGTCCCCAGTACTCTGCATGATTTGCTCCTCAGCACTCGTATCACCAGCGAAGCGG (ssDRT)
AS CRK9^{M501G}	CACACGACCGCTGGCCGCCGTCGGCGCTGCAAGCAAGGCGAAAGATGTTTTTCTGGTG**GGC**GATTATTGCCCATATGATCTTGGGAGCTACATGCGGCGGTACGCGACTGTGGCAGAGCT (ssDRT)
AS CRK9^{M501A}	CACACGACCGCTGGCCGCCGTCGGCGCTGCAAGCAAGGCGAAAGATGTTTTTCTGGTG**GCG**GATTATTGCCCATATGATCTTGGGAGCTACATGCGGCGGTACGCGACTGTGGCAGAGCT (ssDRT)
KKT3$^{D157A-D174A}$	CGTATCACCAGCGAAGCGGAGATGCTTTTCATGGCACATCAAGCCGTACAGGCGGTGTCGTACGTGCACGCAGAGGGCTGCATTCACCGC**GCG**ATCAAGCTTCAGAACTTTGTCTTCGACCTCGA TGGTAATCTGAAGCTGATC**GCG**TTTGGCCTTGCTTGCAATTCCCTAAAACCTCCAGCAGGCGACGTGGTGGCGGGCACTGTGTCTTTCATGTCT (dsDRT)
KKT3$^{D157D-D174D}$	CGTATCACCAGCGAAGCGGAGATGCTTTTCATGGCACATCAAGCCGTACAGGCGGTGTCGTACGTGCACGCAGAGGGCTGCATTCACCGC**GAT**ATCAAGCTTCAGAACTTTGTCTTCGACCTCGA TGGTAATCTGAAGCTGATC**GAT**TTTGGCCTTGCTTGCAATTCCCTAAAACCTCCAGCAGGCGACGTGGTGGCGGGCACTGTGTCTTTCATGTCT (dsDRT)
KKT3^{K64A}	CGCATTGGCCAAGGCTCCTTCGGCACGGTGTACCGCGCCGTCAGCAGCGACTATCCACGTCTCGCGCTG**GCT**ATCTCTACAGGCAAGAGTACGCGGCTTCGCCAGGAGTTAGATGTGCTGAGTCG TGTGTGTACGAAAGGACGG (dsDRT)
KKT3^{K64K}	CGCATTGGCCAAGGCTCCTTCGGCACGGTGTACCGCGCCGTCAGCAGCGACTATCCACGTCTCGCGCTG**AAG**ATCTCTACAGGCAAGAGTACGCGGCTTCGCCAGGAGTTAGATGTGCTGAGTCG TGTGTGTACGAAAGGACGG (dsDRT)
KKT3^{43_327del}	CGTAGCGGCGATTCGGAGATCGTGAAGGAGGTGTGGCCGTGCGAGCGCATT**GGTTCTGGTAGTGGTTCCGGTTCCGGTTCTGGTTCTGGTAGTGGTTCCGGTTCCGGTTCT**ACAATCCTGGAGAA CAAGCTGTGGAACTTACGGCGGCGCTATCCACCTGCA (dsDRT)

AS, analog-sensitive kinase; ssDRT, single-stranded DNA repair template; dsDRT, double-stranded DNA repair template.

Repair template sequence is coloured as follows: homology arm in black, recoded codons in blue, and mutated target codon in red.
