# Supplementary material for: Chemical genetics reveals Leishmania KKT2 and CRK9 kinase activity is required for cell cycle progression
Source: PLoS Pathog. 2026 May 13;22(5):e1014194. doi: 10.1371/journal.ppat.1014194 (PMC13211308; doi:10.1371/journal.ppat.1014194)
Supplement: S4 Table — (PDF) [file ppat.1014194.s004.pdf]

S4 Table – Sequence of oligonucleotides used to screen the CRISPR-Cas9 engineered *L. mexicana* cell lines.

Oligo ID	Engineered cell line	Sequence	Description
OL11917 (F)	CLK1	ATGTCGCGCAGCCAGAGC	Specific amplification of CLK1 to then screen the analog sensitive by additional PCR or restriction site digestion – Sanger sequencing.
OL8401 (R)		GCAGCTCGCTGTGAAAGTAG	
OL11802 (F)	CLK2	AGGAAACCGCTGTGTCAAGCACC	Specific amplification of CLK1 to then screen the analog sensitive or resistant by additional PCR or restriction site digestion – Sanger sequencing.
OL8401 (R)		GCAGCTCGCTGTGAAAGTAG	
OL11592 (F)	AS CLK1^{M213G} / AS CLK2^{M220G}	CCAGAACGACAGCGGCCA	Semi-nested PCR to specifically amplify the wildtype CLK1 or CLK2 genome.
OL8401 (R)		GCAGCTCGCTGTGAAAGTAG	
OL12375 (F)	AS CLK1^{M213G} / AS CLK2^{M220G}	TCAAAATGATTCTGGTCATATGTGCATCGTCG	Semi-nested PCR to specifically amplify the AS CLK1 or AS CLK2 genome.
OL8401 (R)		GCAGCTCGCTGTGAAAGTAG	
OL11617 (F)	AS KKT2^{M146}	CTTCGCGTTAACGTGGATTT	PCR followed by restriction site digestion to screen analog sensitive mutant – Sanger sequencing
OL11618 (R)		TGCAACCTCTGAGACCAGTG	PCR followed by restriction site digestion to screen analog sensitive mutant – Sanger sequencing
OL12165 (R)		CCTCGAGATACGGATTCATGCG	Sanger sequencing
OL11611 (F)	AS KKT3^{M110}<br>KKT3$^{D157A\text{-}D174A}$ / KKT3$^{D157D\text{-}D174D}$<br>KKT3^{K64A} / KKT3^{K64K}<br>KKT3^{43_327del}	CATCGTGCTCTGCACTCTGT	PCR followed by restriction site digestion to screen mutants – Sanger sequencing
OL11612 (R)		TAAGCGACTGTTCAGCAACG	PCR followed by restriction site digestion to screen mutants – Sanger sequencing
OL11605 (F)	AS CRK9^{M501}	CTGCCGGGATGTGAACTACT	PCR followed by restriction site digestion to screen analog sensitive mutant – Sanger sequencing
OL11606 (R)		GCGGTTCCTCCTTGAAGAAT	PCR followed by restriction site digestion to screen analog sensitive mutant – Sanger sequencing
OL14686 (F)	Δ*kkt3*	TCGTGCTCTGCACTCTGTTT	CRISPR-Cas9 Knocked out cell line screening
OL14687 (R)		CCAGCGGGAGGGAGGATAAA	CRISPR-Cas9 Knocked out cell line screening
OL14688 (F)		GTCACAGTGCAGGTGTACGA	CRISPR-Cas9 Knocked out cell line screening
OL14689 (R)		CACGCATGCTCGATCAACAG	CRISPR-Cas9 Knocked out cell line screening
OL12735 (F)	KKT2::mNG::3xMyc<br>KKT2_AS::mNG::3xMyc<br>KKT2_AS::3xMyc::mT	TGCAACGCTATGGTGGACAT	CRISPR-Cas9 endogenous tagged cell line screening
OL12736 (R)		TCATGGTCACGCATCTAGCG	CRISPR-Cas9 endogenous tagged cell line screening
OL14186 (R)		CCTTGCGCTATTCGCTGTTG	CRISPR-Cas9 Knocked out cell line screening – Sanger sequencing
OL14188 (R)		TTGAGGAAGTGCGGTACTGG	PCR followed by restriction site digestion to screen analog sensitive mutant – Sanger sequencing
OL9369 (R)	N/A	GCAGCAGGTCTGCATTATAC	Integration of the DNA repair template in the CRISPR-Cas9 knocked-out cell lines
OL12757 (F)	N/A	GCACAGGTCTCTCAAATTGG	Integration of the DNA repair template in the CRISPR-Cas9 C-terminus endogenous tagged cell lines

R, reverse oligo; F, forward oligo; N/A, not applicable; AS, analog-sensitive kinase; *Δ*, knockout target gene.

Screening PCRs were performed using Platinum Taq DNA Polymerase (Invitrogen, Cat. 10966018) in a 25 µL reaction mix containing 1x PCR buffer, 200 µM dNTPs, 1.5 mM $MgCl_2$, 0.4 µM of each forward and reverse oligos, 6% (v/v) Kb extender and 0.02 U/µL Platinum Taq DNA Polymerase. Cycling condition used was: initial denaturation at 94ºC for 5 minutes followed by 40 cycles of 94ºC for 30 seconds (denaturation), 60ºC for 30 seconds (annealing), 72ºC for 1 minute per Kb (extension) and a final extension step at 72ºC for 10 minutes.
