# Supplementary material for: Chemical genetics reveals Leishmania KKT2 and CRK9 kinase activity is required for cell cycle progression
Source: PLoS Pathog. 2026 May 13;22(5):e1014194. doi: 10.1371/journal.ppat.1014194 (PMC13211308; doi:10.1371/journal.ppat.1014194)
Supplement: S1 Fig — (PDF) [file ppat.1014194.s005.pdf]

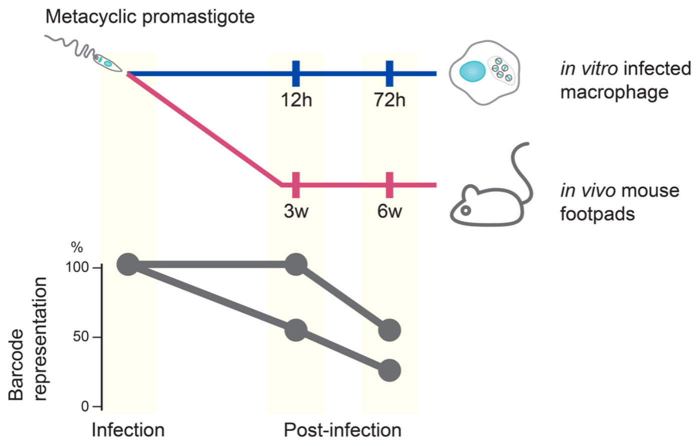

S1 Fig. Schematic illustration to identify protein kinases required for *L. mexicana* amastigote survival. The Bar-Seq dataset generated from the pooled barcoded library of protein kinase deleted mutants [1] was used to identify the protein kinases required for amastigote survival. The proportion of barcodes in the metacyclic promastigote stage and in two post-infection time points – 12 and 72 hours (h) for the *in vitro* infected macrophages, and 3 and 6 weeks (w) for the *in vivo* mouse footpads – was used to calculate fold changes in barcode representation relative to the preceding time point. The line graph shows the trajectories of the barcode representation in a mutant cell line used to classify a protein kinase as required for amastigote survival: (i) a significant ≥50% reduction in barcode abundance at the first post-infection time point, followed by an additional decrease of ≥30% at the second time point; or (ii) no significant change in the first time point, but a significant ≥50% reduction at the final time point. Statistical analysis, comparing the barcode proportion at each time point with those of the preceding time point, was performed by paired Student's t-test.

References

1. Baker N, Catta-Preta CMC, Neish R, Sadlova J, Powell B, Alves-Ferreira EVC, et al. Systematic functional analysis of Leishmania protein kinases identifies regulators of differentiation or survival. Nat Commun. 2021;12(1):1244. Epub 2021/02/25. doi: 10.1038/s41467-021-21360-8. PubMed PMID: 33623024; PubMed Central PMCID: PMCPMC7902614.
