# Supplementary material for: Chemical genetics reveals Leishmania KKT2 and CRK9 kinase activity is required for cell cycle progression
Source: PLoS Pathog. 2026 May 13;22(5):e1014194. doi: 10.1371/journal.ppat.1014194 (PMC13211308; doi:10.1371/journal.ppat.1014194)
Supplement: S2 Fig — (PDF) [file ppat.1014194.s006.pdf]

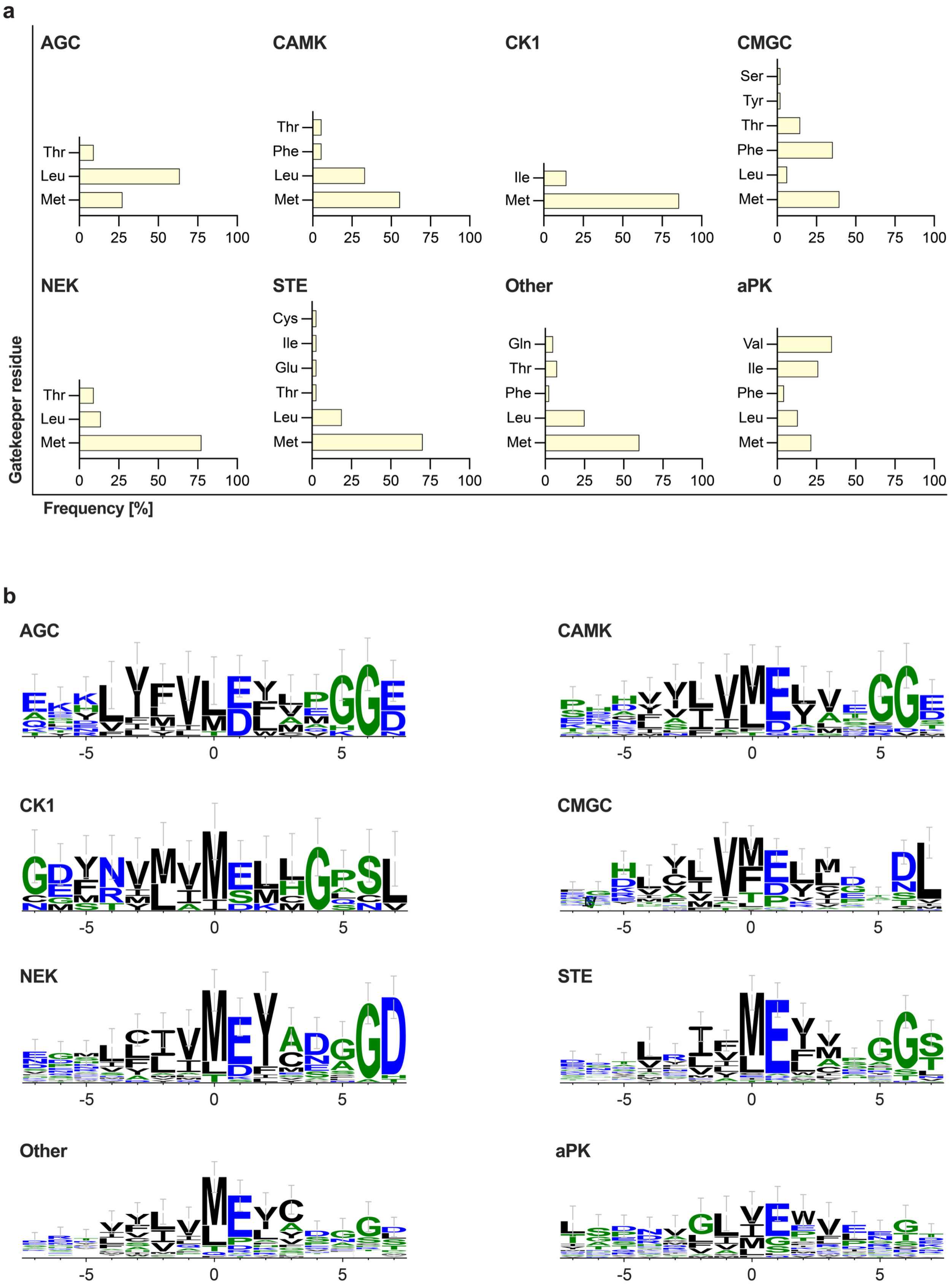

S2 Fig. Gatekeeper residues in the *L. mexicana* kinome. (a) Frequency distribution of gatekeeper residues across kinase groups/families. (b) Amino acid sequence logos depicting the region surrounding the gatekeeper (position 0) for each kinase group/family. The overall height of the stack indicates the level of sequence conservation at that position, while the height of individual symbols within the stack reflects the frequency of each amino acid at that position. Sequence logos were generated using WebLogo 3 [1].

References

1. Crooks GE, Hon G, Chandonia JM, Brenner SE. WebLogo: a sequence logo generator. Genome Res. 2004;14(6):1188-90. doi: 10.1101/gr.849004. PubMed PMID: 15173120; PubMed Central PMCID: PMCPMC419797.
