# Supplementary material for: Chemical genetics reveals Leishmania KKT2 and CRK9 kinase activity is required for cell cycle progression
Source: PLoS Pathog. 2026 May 13;22(5):e1014194. doi: 10.1371/journal.ppat.1014194 (PMC13211308; doi:10.1371/journal.ppat.1014194)
Supplement: S3 Fig — (PDF) [file ppat.1014194.s007.pdf]

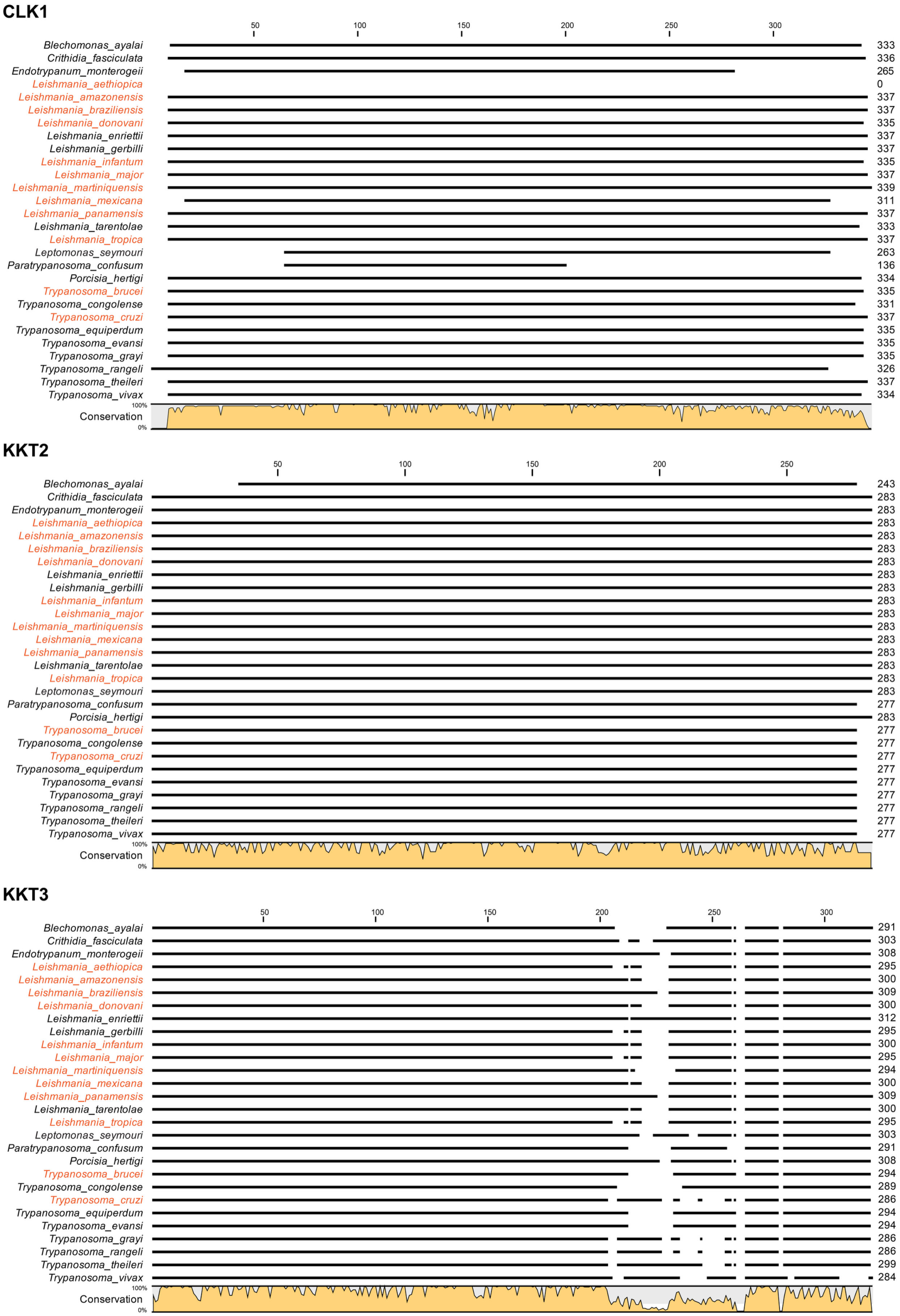

S3 Fig. Conservation of CLK1, KKT2, KKT3 and CRK9 across trypanosomatids.

CRK9

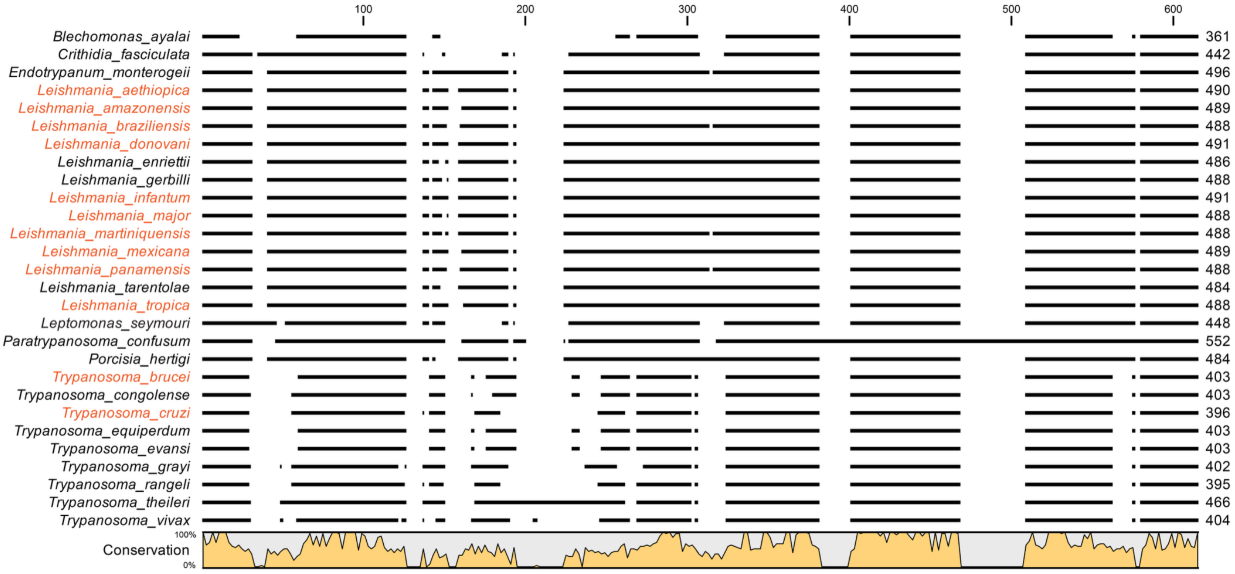

S3 Fig. Conservation of CLK1, KKT2, KKT3 and CRK9 across trypanosomatids. The protein kinase domain of *L. mexicana* CLK1 (LmxM.09.0400), KKT2 (LmxM.36.5350), KKT3 (LmxM.34.4050), and CRK9 (LmxM.27.1940) were aligned with their orthologues from reference trypanosomatid species available in TriTrypDB (https://tritrypdb.org/tritrypdb/app). For *L. donovani*, the CLK1 sequence from strain CL-SL was used due to incomplete sequencing of this gene in the reference strain BPK282A1. Similarly, for *T. cruzi*, KKT3 from the Dm28c 2014 strain was used, as the reference strain CL Brener Esmeraldo-like lacked an annotated KKT3 ortholog. Sequence alignment was performed using the Clustal Omega algorithm in CLC Genomics Workbench v22. The consensus line graph indicating sequence conservation is shown below the alignment. Species known to be human pathogens are highlighted in orange.
