# Supplementary material for: Chemical genetics reveals Leishmania KKT2 and CRK9 kinase activity is required for cell cycle progression
Source: PLoS Pathog. 2026 May 13;22(5):e1014194. doi: 10.1371/journal.ppat.1014194 (PMC13211308; doi:10.1371/journal.ppat.1014194)
Supplement: S4 Fig — (PDF) [file ppat.1014194.s008.pdf]

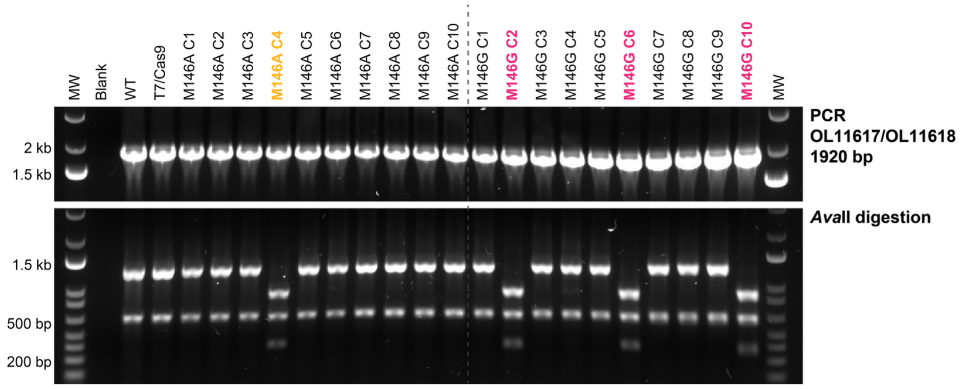

AS	Efficiency
KKT2^{M146G}	30%
KKT2^{M146A}	10%

S4 Fig. CRISPR-Cas9-mediated engineering of analog-sensitive KKT2 in *Leishmania*. Genotypic screening of ten clones (C1 – C10) for each gatekeeper mutation introduced in KKT2. Genotyping results are color-coded as follows: black, wild-type; magenta, KKT2^{M146G}; yellow, KKT2^{M146A}. The editing efficiency for generating analog-sensitive mutants in this experiment is indicated in the lower right corner.
