# Supplementary material for: Chemical genetics reveals Leishmania KKT2 and CRK9 kinase activity is required for cell cycle progression
Source: PLoS Pathog. 2026 May 13;22(5):e1014194. doi: 10.1371/journal.ppat.1014194 (PMC13211308; doi:10.1371/journal.ppat.1014194)
Supplement: S5 Fig — (PDF) [file ppat.1014194.s009.pdf]

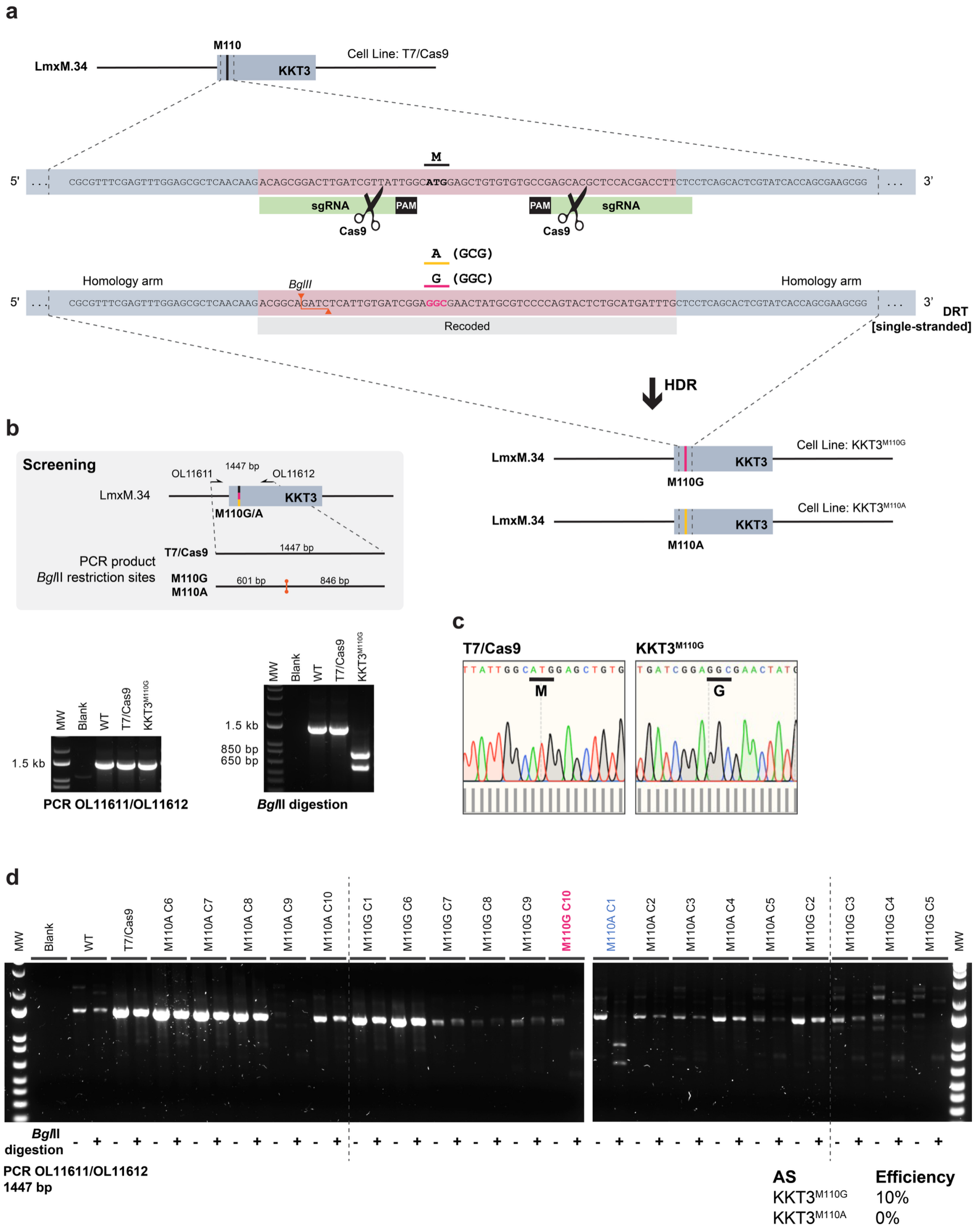

S5 Fig. CRISPR-Cas9-mediated engineering of analog-sensitive KKT3 in *Leishmania*. (a) Schematic of the CRISPR-Cas9 strategy used to engineer analog-sensitive kinases by substituting the KKT3 gatekeeper methionine (M) with glycine (G) or alanine (A). Linear DNA fragments for *in vivo* transcription of two single guide RNAs (sgRNAs), and a 120 bp single stranded DNA repair template (DRT) containing silent recoding mutations and the gatekeeper substitution were used. The mutations introduced a *Bgl*II restriction site, enabling genotypic screening of edited clones. PAM, protospacer adjacent motif; HDR, homology-directed repair. (b) Genotyping workflow (top grey box) and PCR-restriction digest results (bottom) for selected analog-sensitive clones. (c) Sanger sequencing of the engineered KKT3 locus confirms the substitution of the gatekeeper methionine with glycine in the $KKT3^{M110G}$ line. Sequencing chromatograms were visualized in SnapGene v7.2; bar graphs below indicate per-base quality scores. (d)

Genotypic screening of ten clones (C1 – C10) for each gatekeeper mutation introduced in KKT3. Genotyping results are color-coded as follows: black, wild-type; magenta, $KKT3^{M110G}$; blue, $KKT3^{M110M/A}$. The editing efficiency for generating analog-sensitive mutants in this experiment is indicated in the lower right corner.
