# Supplementary material for: Chemical genetics reveals Leishmania KKT2 and CRK9 kinase activity is required for cell cycle progression
Source: PLoS Pathog. 2026 May 13;22(5):e1014194. doi: 10.1371/journal.ppat.1014194 (PMC13211308; doi:10.1371/journal.ppat.1014194)
Supplement: S7 Fig — (PDF) [file ppat.1014194.s011.pdf]

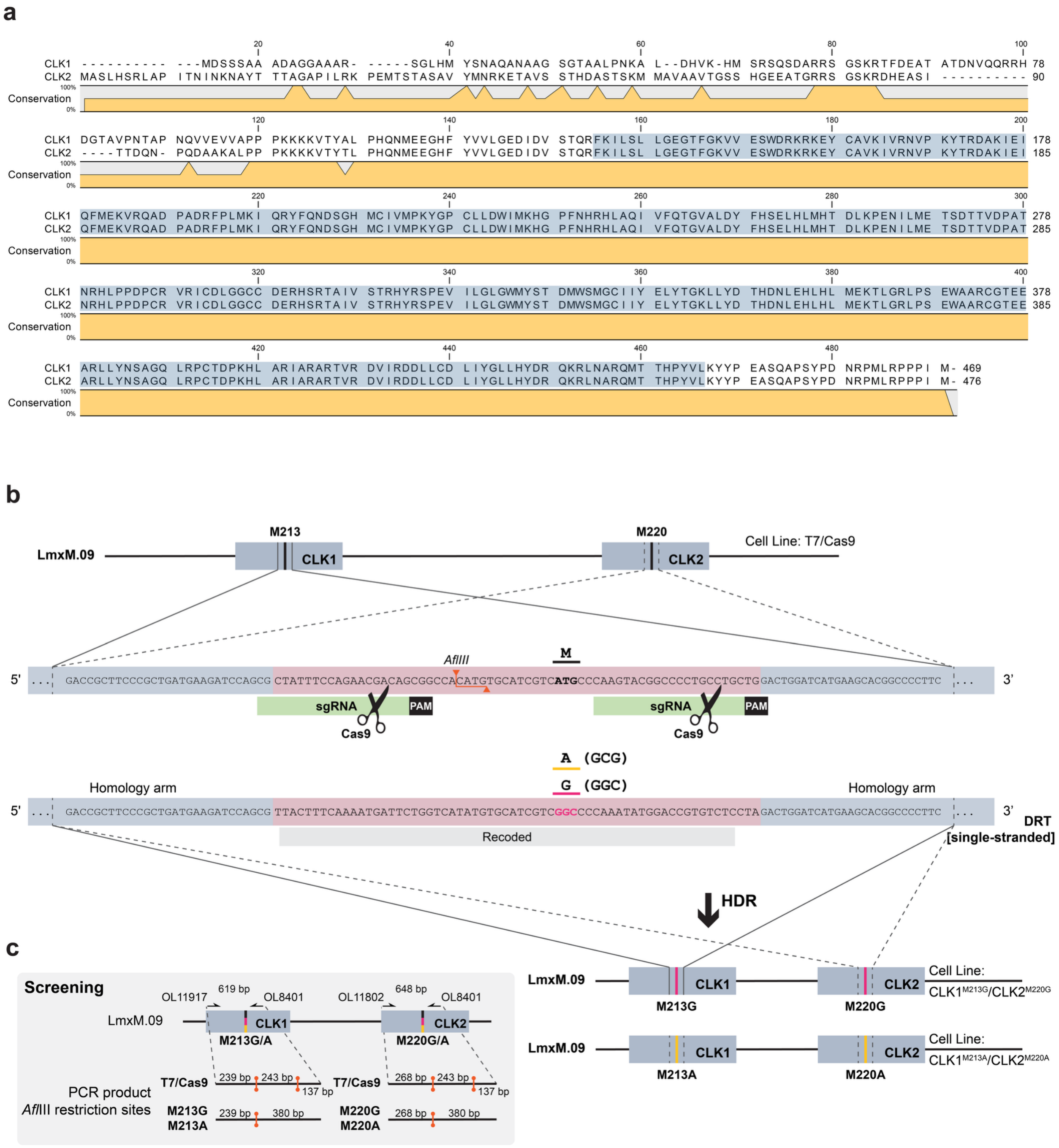

S7 Fig. CRISPR-Cas9-mediated engineering of analog-sensitive CLK1/CLK2 in *Leishmania*.

d

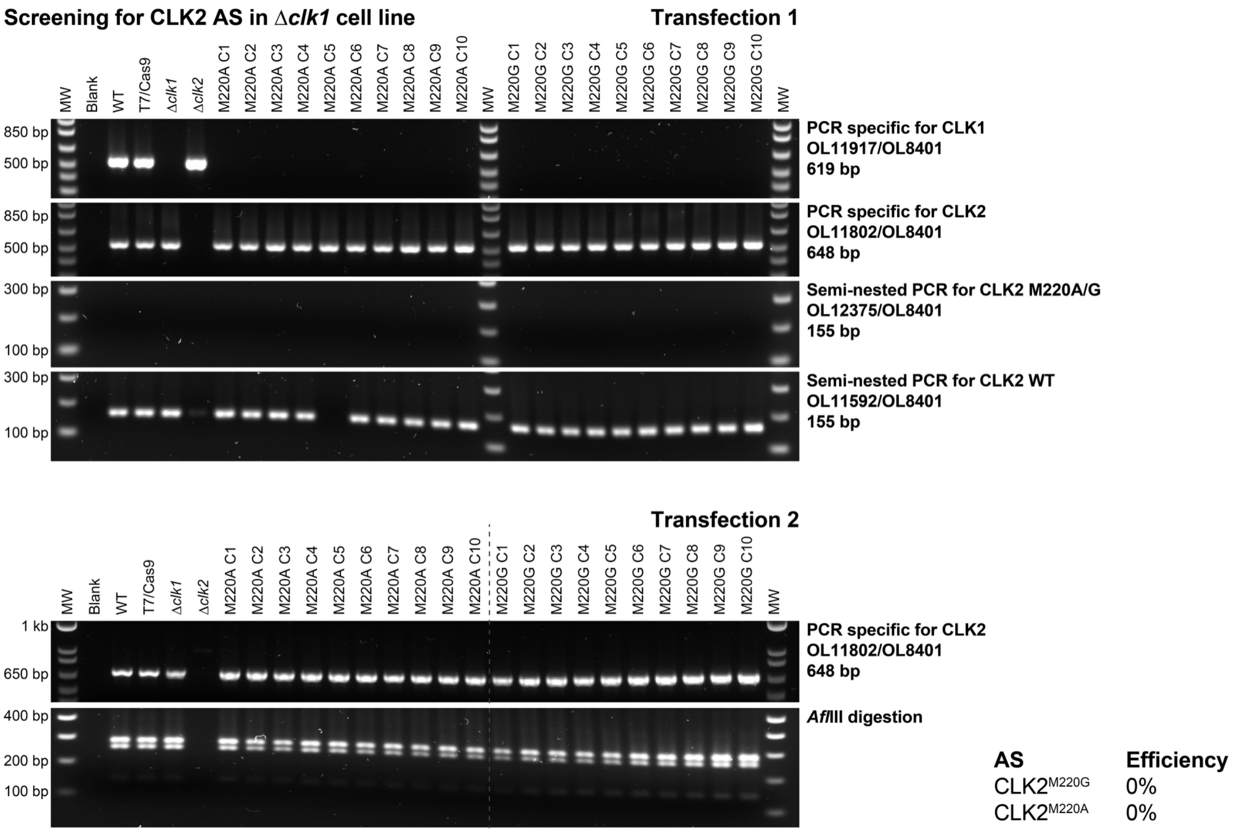

S7 Fig. CRISPR-Cas9-mediated engineering of analog-sensitive CLK1/CLK2 in *Leishmania*.

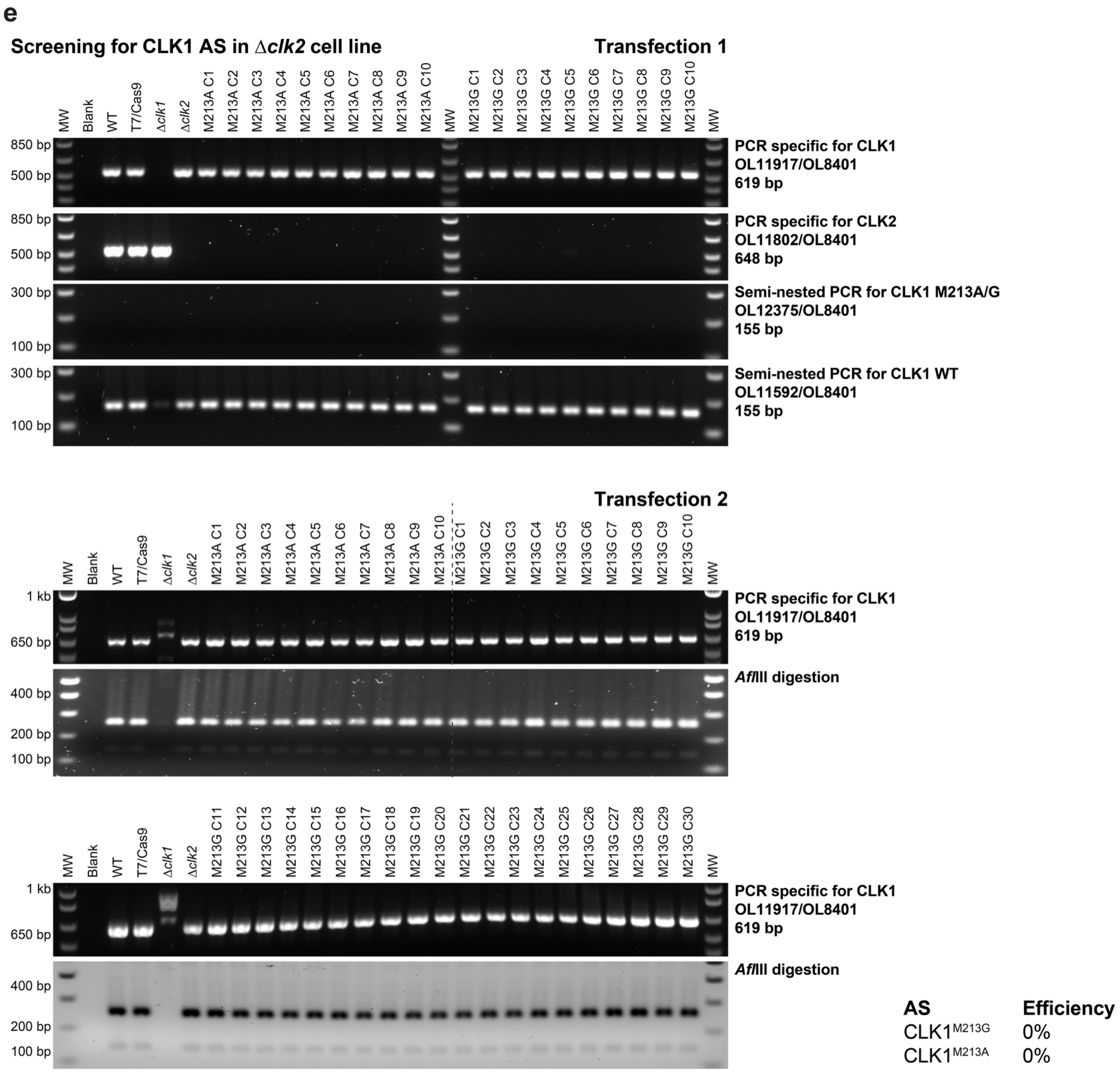

S7 Fig. CRISPR-Cas9-mediated engineering of analog-sensitive CLK1/CLK2 in *Leishmania*.

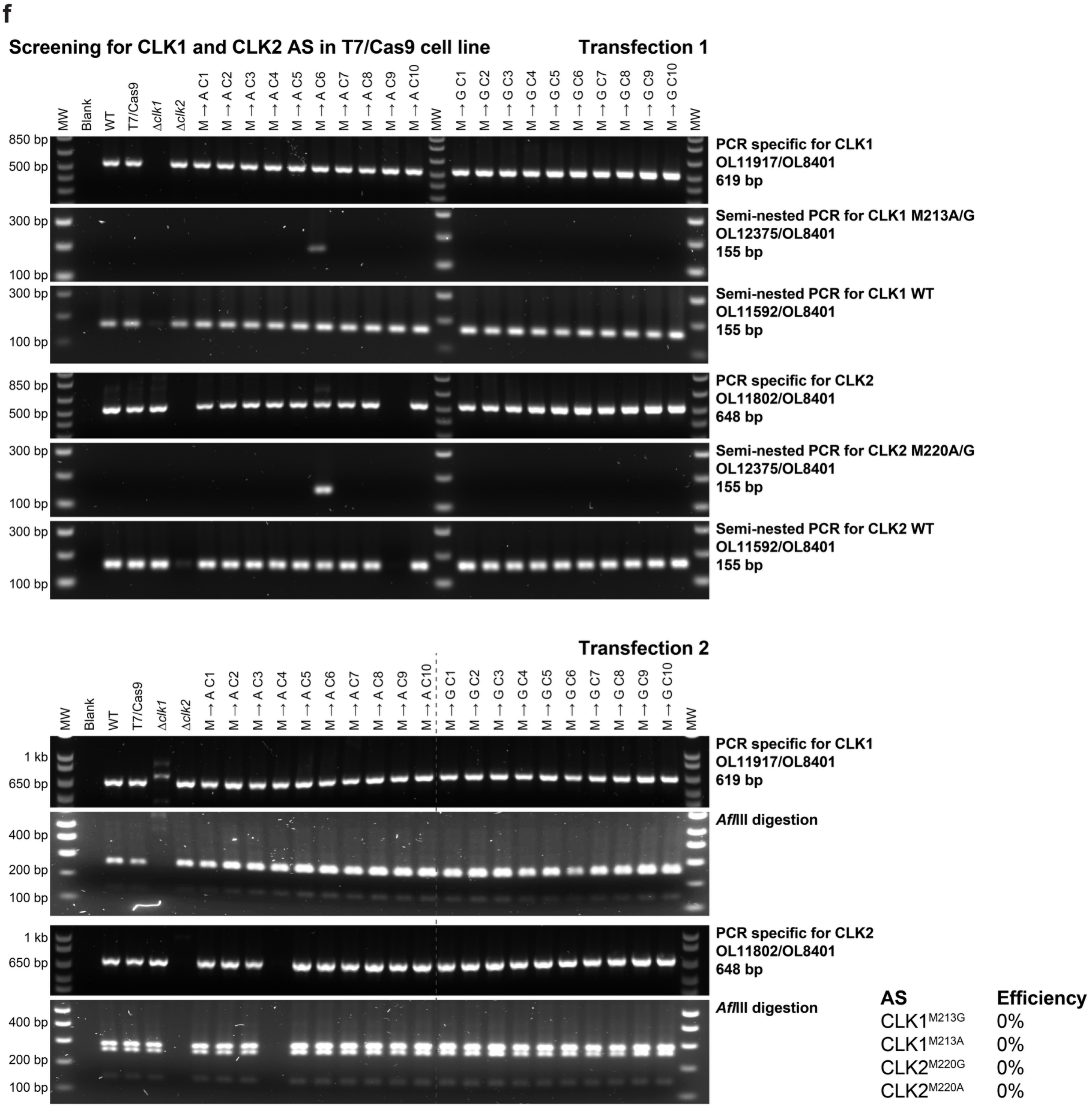

S7 Fig. CRISPR-Cas9-mediated engineering of analog-sensitive CLK1/CLK2 in *Leishmania*. (a) Sequence alignment was performed using the Clustal Omega algorithm in CLC Genomics Workbench v22. The consensus line graph indicating sequence conservation is shown below the alignment. The kinase domains were predicted using InterPro domain analysis [1], and the corresponding amino acid sequences are highlighted in blue. (b) Schematic of the CRISPR-Cas9 strategy used to engineer analog-sensitive kinases by substituting the CLK1 and CLK2 gatekeeper methionine (M) with glycine (G) or alanine (A). Linear DNA fragments for *in vivo* transcription of two single guide RNAs (sgRNAs), and a 120 bp DNA repair template (DRT) containing silent recoding mutations and the gatekeeper substitution were used. The mutations eliminated a *Afl*III restriction site, enabling genotypic screening of edited clones. PAM, protospacer adjacent motif; HDR, homology-directed repair. (c) Genotyping workflow (grey box) used to screen analog-sensitive clones. Genotyping was also performed using a semi-nested PCR: the first PCR was designed over the specific N-terminus for CLK1 (OL11917/OL8401) and CLK2 (OL11802/OL8404) followed by a second PCR that kept the reverse primer (OL8401) and using a specific primer to detect the wildtype genome (OL11592) or the recoded genome (OL12375). (d – e) Genotypic screening of individual clones (C1 – C30) for each gatekeeper mutation attempted in CLK1 and/or CLK2 genes within the *Δclk1*(c) *Δclk2* (d) or T7/Cas9 (e) cell lines. The editing efficiency for generating analog-sensitive mutants is indicated in the lower right corner.

References

1. Paysan-Lafosse T, Blum M, Chuguransky S, Grego T, Pinto BL, Salazar GA, et al. InterPro in 2022. Nucleic Acids Res. 2023;51(D1):D418-D27. doi: 10.1093/nar/gkac993. PubMed PMID: 36350672; PubMed Central PMCID: PMCPMC9825450.
