# Supplementary material for: Chemical genetics reveals Leishmania KKT2 and CRK9 kinase activity is required for cell cycle progression
Source: PLoS Pathog. 2026 May 13;22(5):e1014194. doi: 10.1371/journal.ppat.1014194 (PMC13211308; doi:10.1371/journal.ppat.1014194)
Supplement: S8 Fig — (PDF) [file ppat.1014194.s012.pdf]

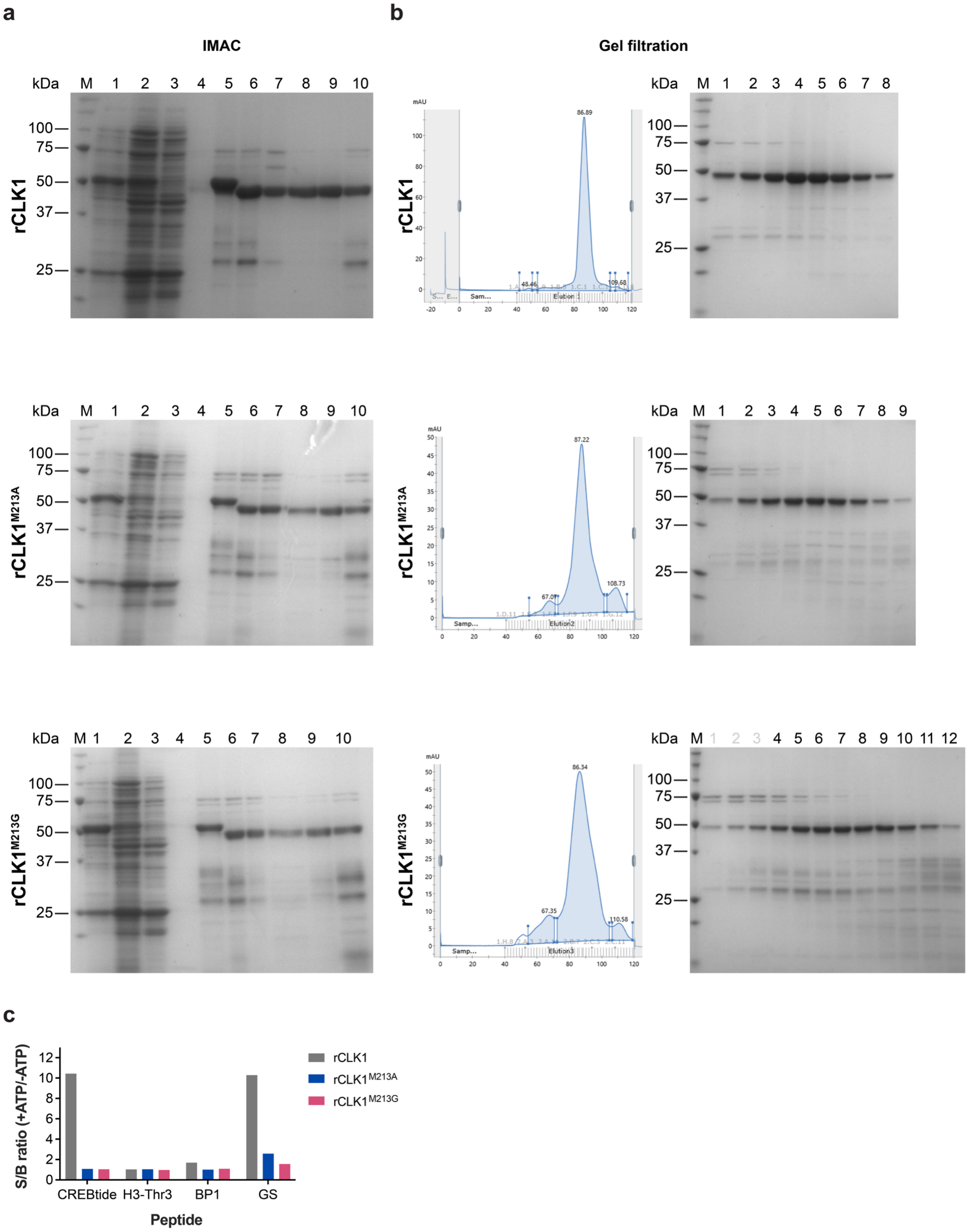

S8 Fig. Purification and substrate recognition of recombinant CLK1 and its analog-sensitive variants. (a) SDS-PAGE analysis of immobilized metal ion affinity chromatography (IMAC) fractions obtained during purification: total lysate [1], supernatant [2], flow-through [3], wash in 30 mM imidazole [4] and eluate in 300 mM imidazole [5]. Following TEV protease cleavage [6], samples were further purified by reverse IMAC on Ni-Sepharose (r-IMAC) and the following fractions were analysed: flow-through [7], wash in 30 mM imidazole [8], wash in 60 mM imidazole [9] and eluate in 300 mM imidazole [10]. (b) Gel filtration chromatograms of TEV-cleaved proteins from the reverse IMAC step (fractions 7 and 8, flow-through and wash in 30 mM imidazole, respectively) (left panel), and SDS-PAGE analysis of the corresponding fractions (right panel). Fractions 1, 2 and 3 (grey) were not included in the purified

protein pool for further analysis. M, molecular weight marker (Precision Plus Protein Unstained Protein Standards, Bio-Rad). (c) Substrate recognition by recombinant CLK1 and its analog-sensitive variants was assessed using an *in vitro* kinase assay (LANCE Ultra Kinase Assay, Perking Elmer) with peptide substrates ULight-CREBtide (Ser133) (#TRF0107), ULight-Histone H3 (Thr3) (#TRF0125), ULight-4E-BP1 (#TRF0128), and ULight-GS (#TRF0131), each paired with its corresponding antibodies. Signal-to-background (S/B) ratio (+ATP/-ATP) is shown. CREBtide peptide (sequence: CKRREILSRRPSYRK) is a synthetic peptide derived from human cAMP Response Element Binding (CREB) protein (phosphorylation site: Ser133). Histone H3 peptide (sequence: ARTKQTARKSTGGK) is derived from residues 463-480 of human RAC-alpha serine/threonine-protein kinase (AKT1/PKB). Phospho-4E-BP1 is a synthetic peptide containing residues surrounding Thr37 and Thr46 of human eukaryotic translation initiation factor 4E-binding protein 1 (phosphorylation motif: STTPGGTLFSTTPG). Glycogen synthase peptide (sequence: PASVPPSPSLSRHSSPHQ(pS)ED) is a synthetic peptide containing residues surrounding Ser641 and Ser657 of human muscle Glycogen Synthase.
