# Supplementary material for: Chemical genetics reveals Leishmania KKT2 and CRK9 kinase activity is required for cell cycle progression
Source: PLoS Pathog. 2026 May 13;22(5):e1014194. doi: 10.1371/journal.ppat.1014194 (PMC13211308; doi:10.1371/journal.ppat.1014194)
Supplement: S9 Fig — (PDF) [file ppat.1014194.s013.pdf]

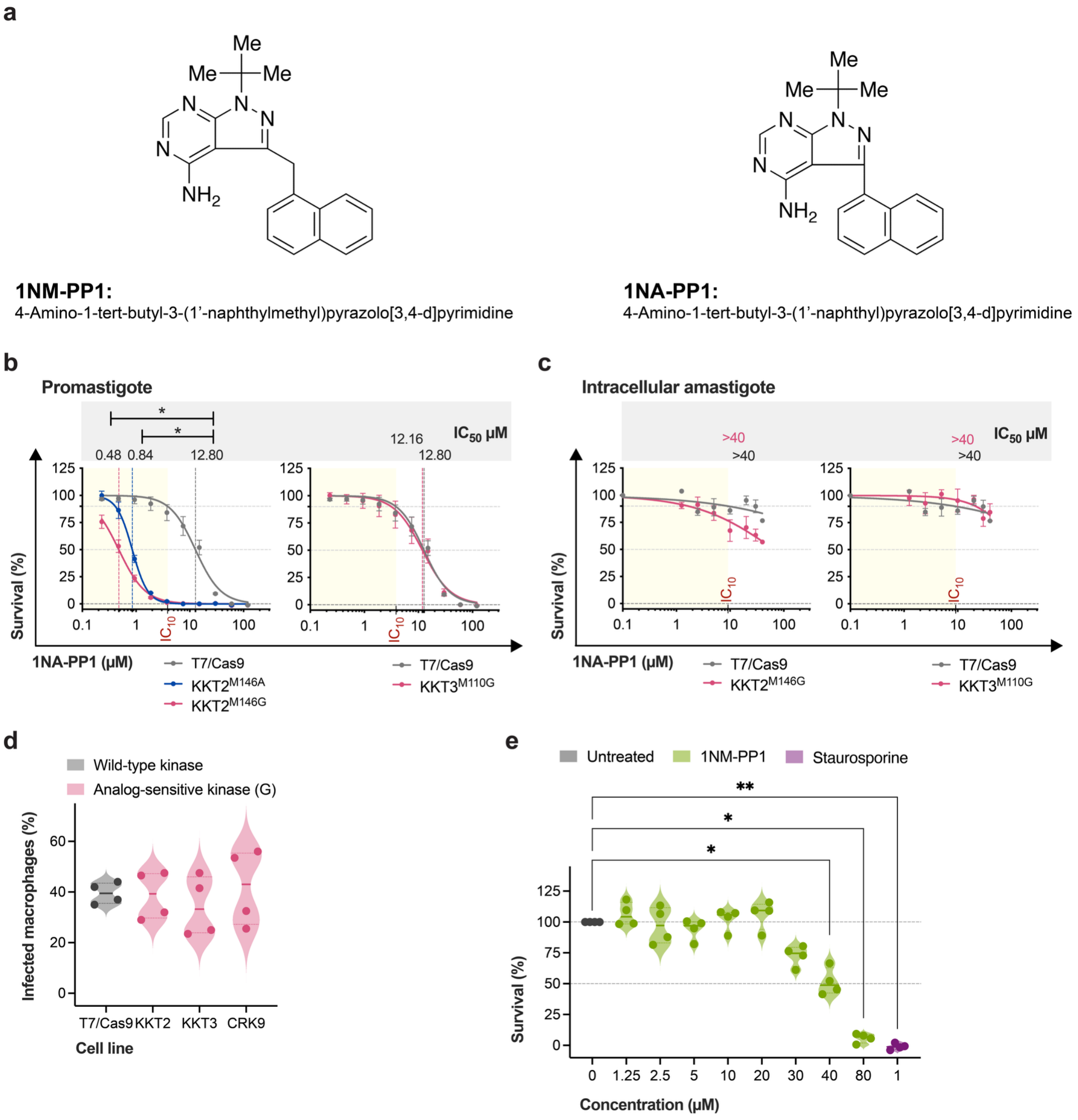

S9 Fig. Susceptibility of *L. mexicana* lines expressing analog-sensitive variants of KKT2 and KKT3 to 1NA-PP1. (a) Chemical structures of the bumped kinase inhibitors used in this study. Parasite viability under 1NA-PP1 treatment was assessed in *L. mexicana* analog-sensitive lines and wild-type T7/Cas9. (b-c) Dose-response curves were fitted using GraphPad Prism v10.4.1, with viability normalized to untreated controls (set at 100% for each cell line). Statistical significance was evaluated using unpaired two-tailed Student's t-tests (*p-value <0.05). The IC_{10} value denotes the concentration of 1NA-PP1 that reduces the viability of the wild-type T7/Cas9 line by 10%. (b) Susceptibility of promastigotes to 1NA-PP1 was measured using a resazurin-based viability assay. Data represent mean ± SEM from three biological replicates. (c) Susceptibility of intracellular amastigotes to 1NA-PP1 was assessed by quantifying the percentage of infected macrophages. Data represent mean ± SEM from four biological replicates. Susceptibility of *L. mexicana* lines expressing AS variants of CRK9 was previously published by Jones N.G. et al., 2023 [1]. (d) Infectivity/proliferation. The percentage of infected macrophages under untreated conditions, obtained from the intracellular amastigote susceptibility assay, was used to estimate infectivity/proliferation of the analog-sensitive kinase lines harbouring a glycine gatekeeper mutation in comparison with the parental T7/Cas9 control. Statistical significance was evaluated using unpaired two-tailed Student's t-tests. No statistically significant differences were observed. (e) Cytotoxicity of the bumped kinase inhibitor 1NM-PP1 in uninfected bone marrow-derived macrophages (BMDM). Cell toxicity was assessed using a resazurin-based assay. Data represent the mean ± SEM from four biological replicates. Statistical significance was assessed using the nonparametric Kruskal-Wallis test, with each treatment group compared to the untreated control (*p-value <0.05, **p-value <0.01).

References

1. Jones NG, Geoghegan V, Moore G, Carnielli JBT, Newling K, Calderon F, et al. Bromodomain factor 5 is an essential regulator of transcription in Leishmania. Nat Commun. 2022;13(1):4071. Epub 20220713. doi: 10.1038/s41467-022-31742-1. PubMed PMID: 35831302; PubMed Central PMCID: PMCPMC9279504.
