# Supplementary material for: Chemical genetics reveals Leishmania KKT2 and CRK9 kinase activity is required for cell cycle progression
Source: PLoS Pathog. 2026 May 13;22(5):e1014194. doi: 10.1371/journal.ppat.1014194 (PMC13211308; doi:10.1371/journal.ppat.1014194)
Supplement: S10 Fig — (PDF) [file ppat.1014194.s014.pdf]

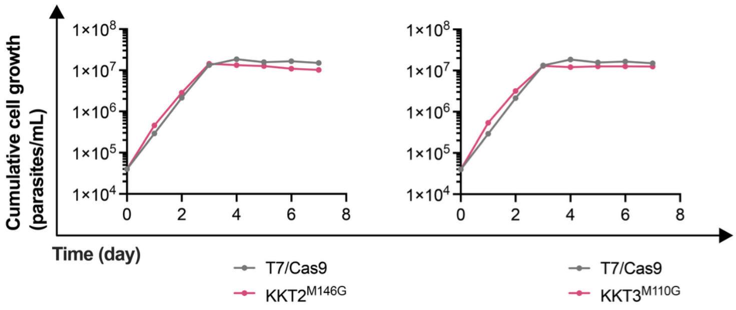

S10 Fig. Growth curves of *L. mexicana* analog-sensitive kinase mutants and parental line. Growth kinetics of *L. mexicana* promastigotes were assessed for the parental T7/Cas9 line and analog-sensitive kinase mutant lines ($KKT2^{M146G}$ and $KKT3^{M110G}$). Growth curve of *L. mexicana* line expressing AS variants of $CRK9^{M501G}$ was previously published by Jones N.G. et al., 2023 [1]. Cultures were initiated at a density of $4x10^4$ cells mL^{-1} in HOMEM medium supplemented with 10% heat-inactivated fetal bovine serum, and cumulative cell densities were measured daily by manual counting using a Neubauer chamber. Growth rates were calculated from the logarithmic phase of the growth curve (0 – 96 h) and are reported as mean ± SEM: T7/Cas9, 1.53 ± 0.007; $KKT2^{M146G}$, 1.45 ± 0.008; $KKT3^{M110G}$, 1.43 ± 0.022; and $CRK9^{M501G}$, 1.43 ± 0.006. Statistical comparisons between mutants and the parental T7/Cas9 line were performed using an unpaired two-tailed Student's t-test, and no significant differences in growth rates were observed.

References

1. Jones NG, Geoghegan V, Moore G, Carnielli JBT, Newling K, Calderon F, et al. Bromodomain factor 5 is an essential regulator of transcription in Leishmania. Nat Commun. 2022;13(1):4071. Epub 20220713. doi: 10.1038/s41467-022-31742-1. PubMed PMID: 35831302; PubMed Central PMCID: PMCPMC9279504.
