# Supplementary material for: Chemical genetics reveals Leishmania KKT2 and CRK9 kinase activity is required for cell cycle progression
Source: PLoS Pathog. 2026 May 13;22(5):e1014194. doi: 10.1371/journal.ppat.1014194 (PMC13211308; doi:10.1371/journal.ppat.1014194)
Supplement: S11 Fig — (PDF) [file ppat.1014194.s015.pdf]

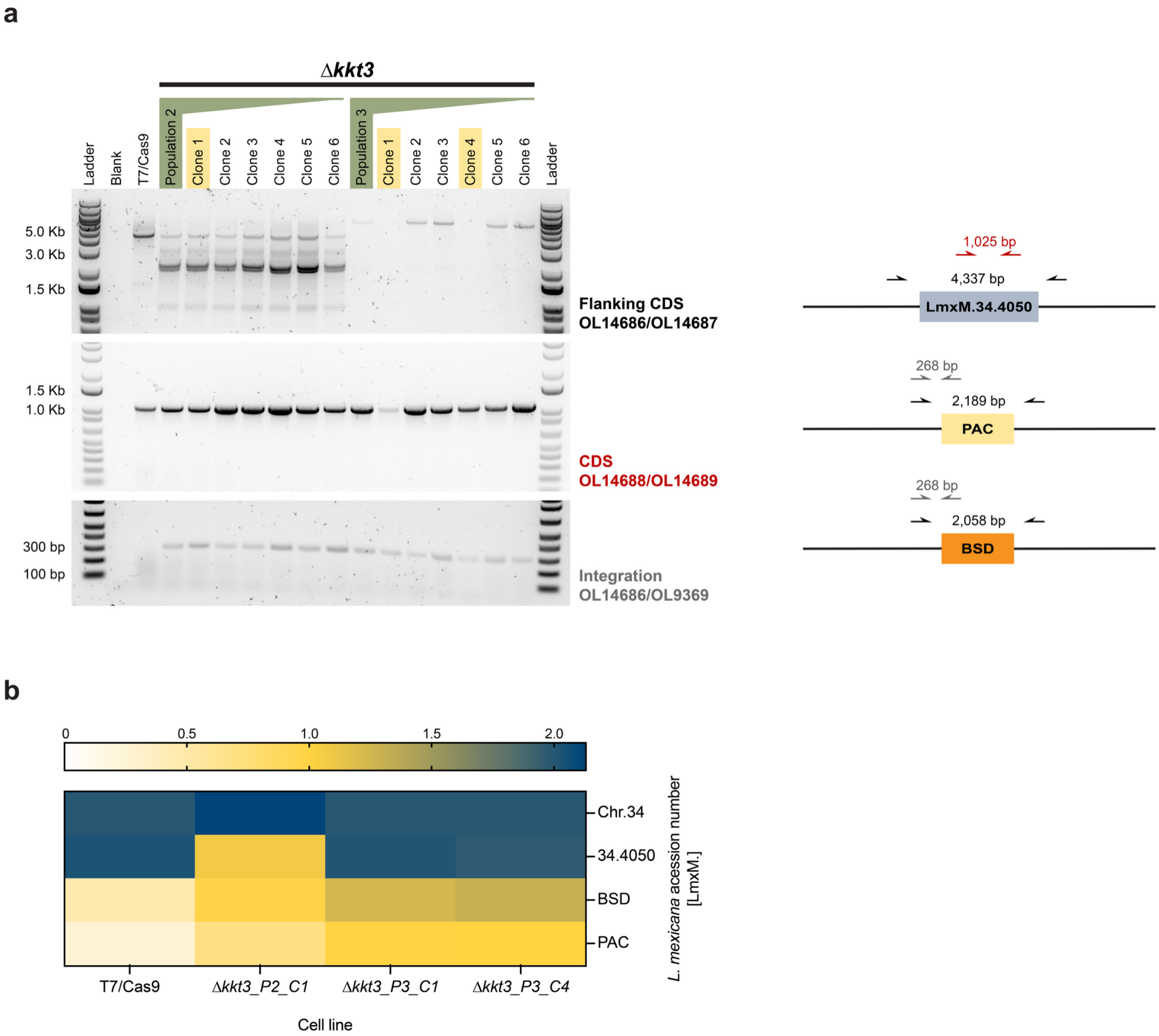

S11 Fig. Requirement of KKT3 protein kinase for *L. mexicana* promastigote survival. Three independent CRISPR-Cas9 transfections were performed to delete the KKT3 gene (LmxM.34.4050) using repair templates carrying blasticidin (*BSD*) and puromycin (*PAC*) resistance markers in combination with linear DNA fragments for *in vivo* transcription of two specific single guide RNAs. Following drug selection, viable populations emerged from transfections 2 and 3, and clones were subsequently isolated. (a) PCR amplification was conducted to assess the presence of the *KKT3* coding sequence (CDS) and integration of the drug resistance cassettes. The PCR strategy and expected amplicon sizes are illustrated in the schematic on the right. Clones highlighted in yellow were selected for whole-genome sequencing (WGS) using Illumina technology. (b) WGS analysis of Δ*kkt3* mutants. Illumina reads from clone 1 of population 2 (P2_C1), and clones 1 and 4 of population 3 (P3_C1 and P3_C4), were aligned to the reference genome of the T7/Cas9 parental cell line [1]. The heatmap shows the copy-number status of chromosome 34 and the *KKT3* locus. Chromosome ploidy was estimated by normalizing read depth to the average coverage of the four longest disomic chromosomes (Chr.08, Chr.20, Chr.33 and Chr.34), which were set to a baseline of 2. Gene copy number was estimated by the ratio of the gene coverage by its chromosome coverage, multiplied by the chromosome ploidy. WGS data for all analysed mutants have been deposited in the SRA under project ID PRJNA1303394.

References

1. Beneke T, Dobramysl U, Catta-Preta CMC, Mottram JC, Gluenz E, Wheeler RJ. Genome sequence of Leishmania mexicana MNYC/BZ/62/M379 expressing Cas9 and T7 RNA polymerase. Wellcome Open Res. 2022;7:294. Epub 20230223. doi: 10.12688/wellcomeopenres.18575.2. PubMed PMID: 36874584; PubMed Central PMCID: PMCPMC9975418.
