# Supplementary material for: Chemical genetics reveals Leishmania KKT2 and CRK9 kinase activity is required for cell cycle progression
Source: PLoS Pathog. 2026 May 13;22(5):e1014194. doi: 10.1371/journal.ppat.1014194 (PMC13211308; doi:10.1371/journal.ppat.1014194)
Supplement: S12 Fig — (PDF) [file ppat.1014194.s016.pdf]

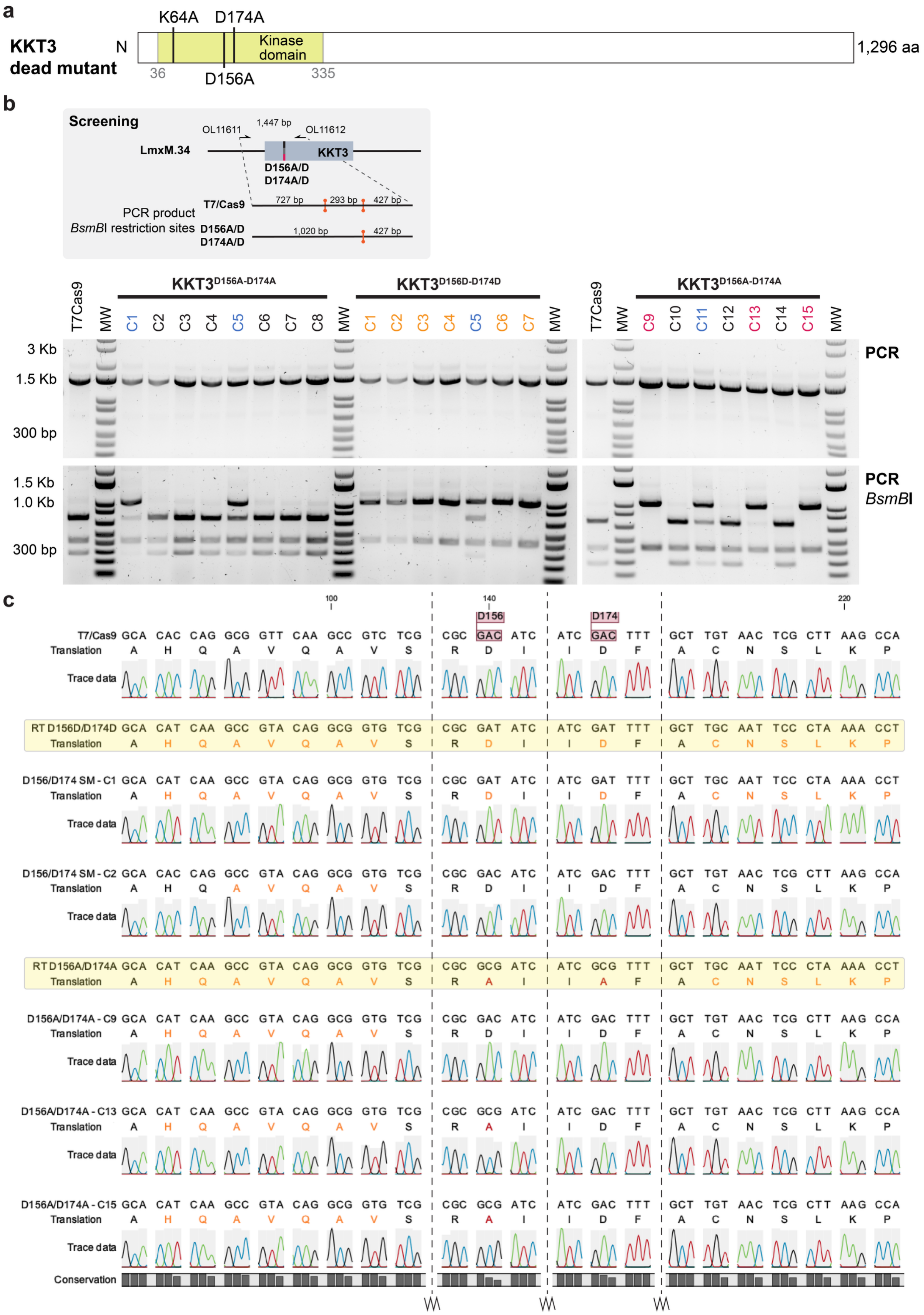

S12 Fig. Engineering KKT3 kinase-dead mutants via targeted mutation of the three catalytic residues.

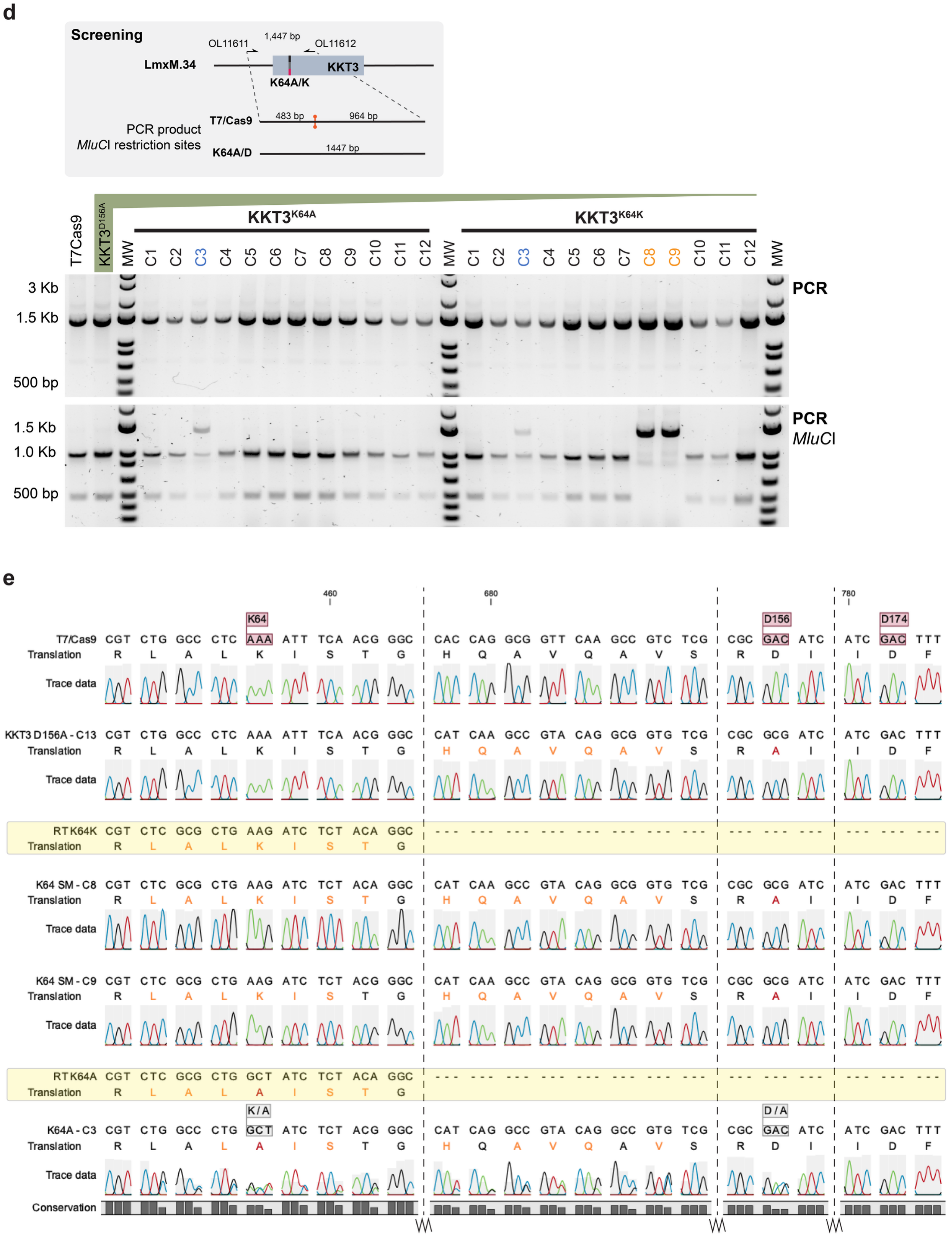

S12 Fig. Engineering KKT3 kinase-dead mutants via targeted mutation of the three catalytic residues. (a) Schematic of the KKT3 protein showing the kinase domain and the three key catalytic residues (K64, D156 and D174). Precise genome editing by CRISPR-Cas9 using a double-stranded DNA repair template was employed to substitute the indicated residues with alanine. (b) Genotyping strategy (top, grey box) and PCR-restriction digest results (bottom) of selected clones following attempts to introduce simultaneous alanine substitutions at D156 and D174. Genotypes are colour-coded as follows: black, wild type; magenta, repair template carrying the alanine substitution at the targeted residues integrated in both alleles; yellow, repair template carrying a silent mutation at the

target residues integrated in both alleles; blue, repair template carrying the alanine or synonymous mutations integrated heterozygously. (c) Sanger sequencing of the edited KKT3 locus confirms integration of repair templates carrying either alanine substitutions or silent mutations (SM) at D156 and D174 in both alleles; however, substitution of the DFG aspartate with alanine was not detected in any screened clone. Sequencing chromatograms were visualized in CLC Main Workbench 22; background grey bar plots indicate per-base quality scores. (d) Genotyping strategy (top, grey box) and PCR-restriction digest results (bottom) of selected clones following targeting of K64 for alanine substitution in the KKT3^{D156A} cell line. Genotypes are colour-coded as follows: black, wild type; yellow, repair template carrying a silent mutation integrated in both alleles; blue, repair template carrying either the alanine substitution or the synonymous mutation integrated heterozygously. (e) Sanger sequencing of the edited KKT3 locus confirms homozygous integration of the repair template carrying the silent mutation (SM), whereas the alanine substitution at K64 was detected only in a heterozygous configuration. Sequencing chromatograms were visualized in CLC Main Workbench 22; background grey bar plots indicate per-base quality scores.
