# Supplementary material for: Chemical genetics reveals Leishmania KKT2 and CRK9 kinase activity is required for cell cycle progression
Source: PLoS Pathog. 2026 May 13;22(5):e1014194. doi: 10.1371/journal.ppat.1014194 (PMC13211308; doi:10.1371/journal.ppat.1014194)
Supplement: S13 Fig — (PDF) [file ppat.1014194.s017.pdf]

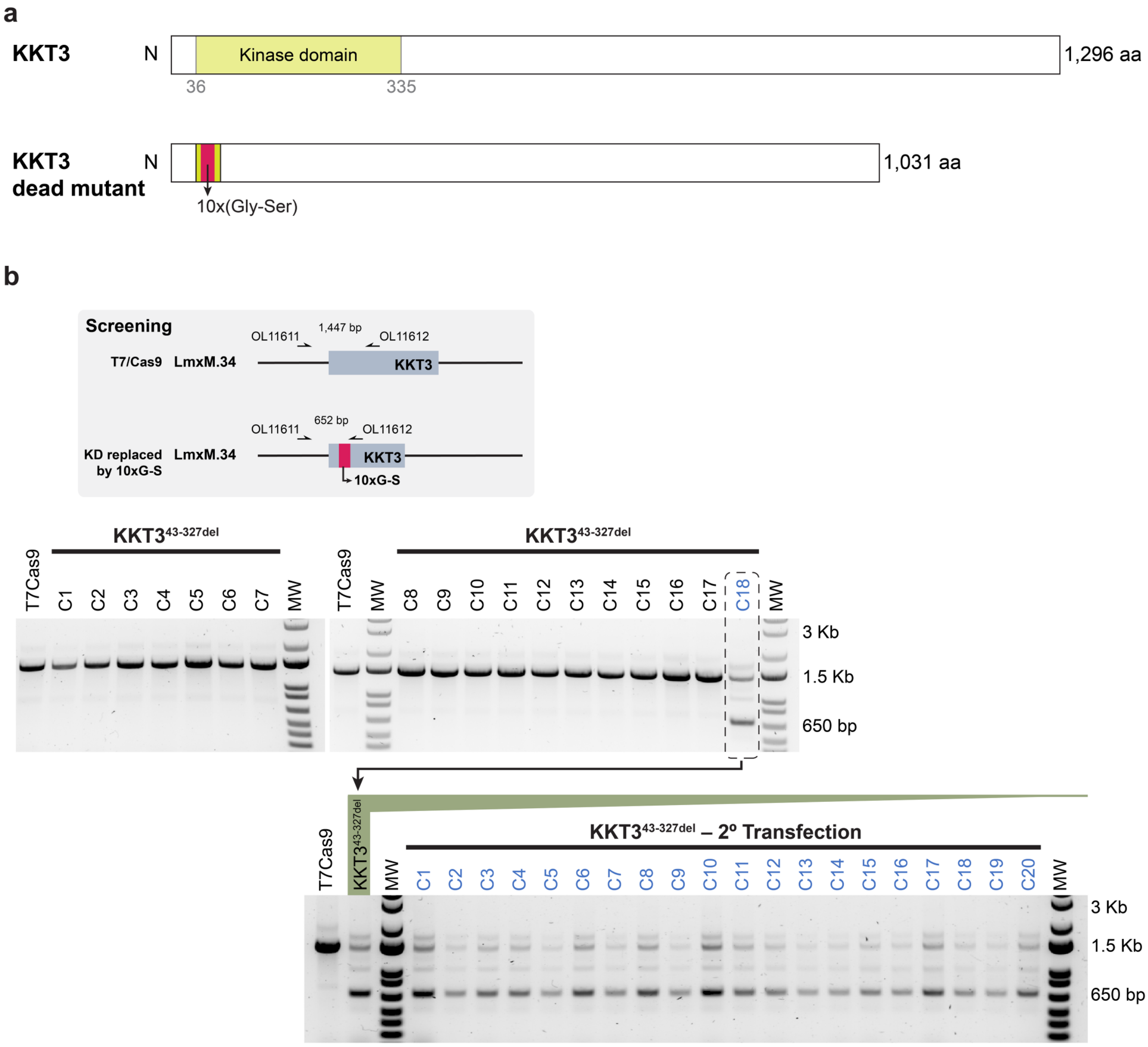

S13 Fig. Engineering KKT3 kinase-dead mutants by deletion of the kinase domain. (a) Schematic of the KKT3 protein showing the kinase domain in the parental line (top) and its replacement with a 10x(Gly-Ser) linker in the attempted mutant (bottom). Insertion of the linker maintains the reading frame, preserving all downstream protein sequences. (b) Genotyping strategy (top, grey box) and PCR analysis (bottom) of selected clones. A second round of transfection was performed in the single heterozygous clone recovered after the initial transfection (2º Transfection). Genotypes are colour-coded as follows: black, wild type; blue, heterozygous.
