# Supplementary material for: Chemical genetics reveals Leishmania KKT2 and CRK9 kinase activity is required for cell cycle progression
Source: PLoS Pathog. 2026 May 13;22(5):e1014194. doi: 10.1371/journal.ppat.1014194 (PMC13211308; doi:10.1371/journal.ppat.1014194)
Supplement: S14 Fig — (PDF) [file ppat.1014194.s018.pdf]

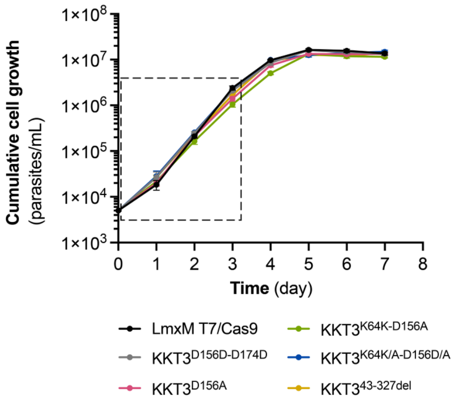

S14 Fig. Growth kinetics of *L. mexicana* KKT3 kinase dead mutants and parental line. (a) Growth curves of *L. mexicana* promastigotes were assessed for the parental T7/Cas9 line and KKT3 kinase dead mutants. Cultures were initiated at a density of 5x10^3 cells mL^{-1} in HOMEM medium supplemented with 10% heat-inactivated fetal bovine serum, and cumulative cell densities were measured daily by manual counting using a Neubauer chamber. The boxed region highlights the logarithmic phase of growth (0 – 72 h), which was used for growth rate calculations.
