# Supplementary material for: Chemical genetics reveals Leishmania KKT2 and CRK9 kinase activity is required for cell cycle progression
Source: PLoS Pathog. 2026 May 13;22(5):e1014194. doi: 10.1371/journal.ppat.1014194 (PMC13211308; doi:10.1371/journal.ppat.1014194)
Supplement: S15 Fig — (PDF) [file ppat.1014194.s019.pdf]

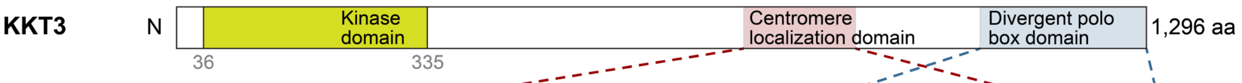


```
LmxM   LGLDVIWQDE ADHRRTLSAM LLIEHAWLLA SFRLTIEEDQ ERYNITWLAE EQEKSAAHPH RFKEVMQVIS KKYQYGFVCD
Tb927  SGF---VTME NAVRNVKTSL LLLEHSCMVT RLSTVVTELF DREHMVWLEK EQRKSATHPH TFREM-GRAN KKYRYGFVCD
        *:        *      *.. :::  **:**: :::   :   .: *  :* ::.** :  **.***:***  *:*:      .  ***:******

LmxM   MCDYEFLPTG PGEKDLHFFH CPCGRDLCPD CYTAYQRQCT CSCCRVVHSN SCVLREHLLR TGGTQYYSGS
Tb927  VCCFEFEPVG S---SMYFFH CQCGRDMCPK CYEEYANNYT CDACGREFAS SGALRRHSCS C--------V
       :* :** *.*      .::*** * ****:**. **   *  .: *  *..*    .:.  *  .**.*

LmxM   PEERDALLNG EWIRHFYLFP HAEREHGAAS GTWAEGEEEP YAFVYHAQPG RTGAIFLTSD FPMHSAVFSM LERQFFVVNQ
Tb927  PEERHMLLNG DWIRYYHFYP MEEEG----- -------GDS VAVTYHIQPG RTGVTFFNHS FSVHSAVLSV LEHIVYVVDR
       ****. ****  :***:::::*   *.                  :   *..** ***  ***. *:. .  * :****:*:  **:  .:**::

LmxM   VDTVEGADST RATSLLKAKG HPELRIAFHA LQDIVAYDTN MMKQQRTPGT VSVYQAPRSA YSCNGEPFLY VRWFRFNENR
Tb927  VDIEEDNDVA RILSLAQAL- -NEEKKIYDV LQLVETHDTH MLKQRRSPGI MSVYCPPQTA FQCNGDPFVF VRWYRFHMEN
       **   *. * :  *   ** :*      *  :  :..  ** : ::**:  *:**:*:**  :***   *::*  :.***:**::  ***:**: :.

LmxM   TLSAFLLSNG AVQVFVNNEY ELRWFDESRK FLIRYNGVCE LVDDGTFALA PGINHLLYDS FDA
Tb927  SMSGFMLSNG AVQVFVGGKY ELRWLDDNRK FIVRSNGVCE VLDEEKFPLS EELNQMLYGG V--
       ::*.*:****  ******..:*  ****:*:.**  *::* *****  ::*:  .* *:    :*::**..  .
```

LmxM.KKT3 / Tb927.KKT3 Identity	Full length	Kinase domain	Centromere localization domain	Divergent polo box domain
	44.27%	59.86%	40.00%	48.31%

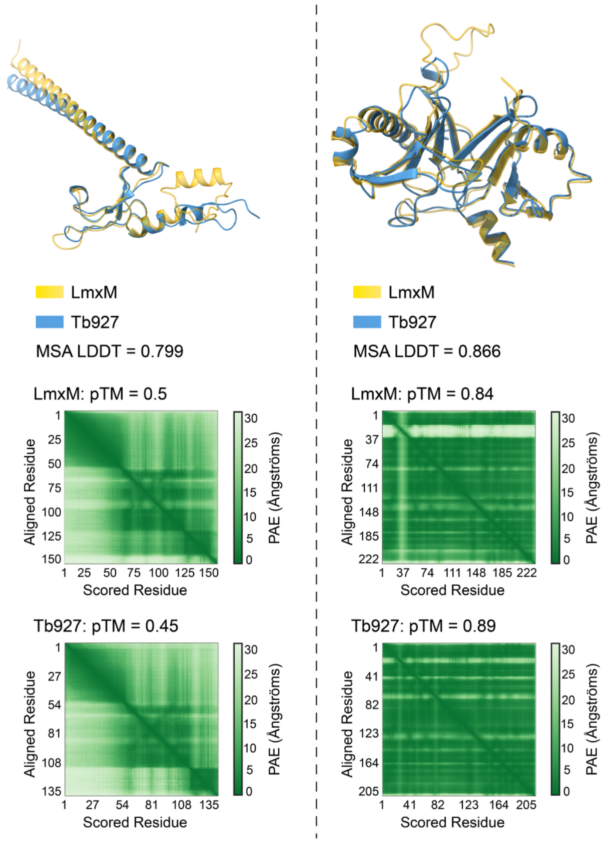

S15 Fig. Domain architecture of *L. mexicana* KKT3. Protein sequence alignment using *T. brucei* (Tb927) KKT3 as a reference identified regions in *L. mexicana* (LmxM) KKT3 corresponding to the previously described centromere localization domain and divergent polo box domain. Sequence identity between *L. mexicana* and *T. brucei* KKT3 for the full-length protein and individual domains is summarized in the table. Structural models of the centromere localization and divergent polo box domains from *L. mexicana* and *T. brucei* KKT3 were generated using AlphaFold 3 [1] and visualized in ChimeraX v1.9. Structural alignments were performed using Foldseek [2] online tool, and similarity was quantified using the multiple-sequence-alignment local distance difference test (MSA LDDT) score. AlphaFold confidence metrics, including the predicted template modelling score (pTM), together with the Foldseek MSA LDDT values, are shown below the aligned structural models.

References

1. Abramson J, Adler J, Dunger J, Evans R, Green T, Pritzel A, et al. Accurate structure prediction of biomolecular interactions with AlphaFold 3. Nature. 2024;630(8016):493-500. Epub 20240508. doi: 10.1038/s41586-024-07487-w. PubMed PMID: 38718835; PubMed Central PMCID: PMCPMC11168924.

2. Gilchrist CLM, Mirdita M, Steinegger M. Multiple Protein Structure Alignment at Scale with FoldMason. bioRxiv. 2024:2024.08.01.606130. doi: 10.1101/2024.08.01.606130.
