# Supplementary material for: Chemical genetics reveals Leishmania KKT2 and CRK9 kinase activity is required for cell cycle progression
Source: PLoS Pathog. 2026 May 13;22(5):e1014194. doi: 10.1371/journal.ppat.1014194 (PMC13211308; doi:10.1371/journal.ppat.1014194)
Supplement: S17 Fig — (PDF) [file ppat.1014194.s021.pdf]

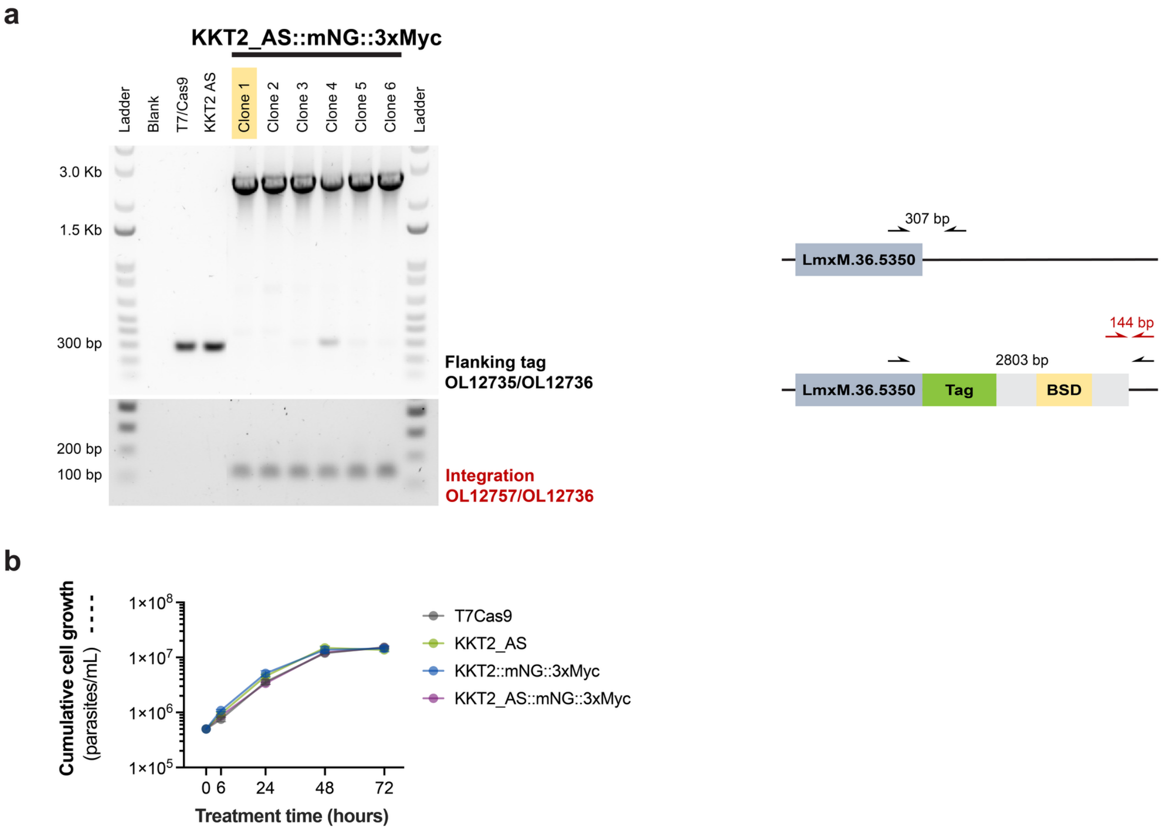

S17 Fig. Engineering of the KKT2_AS::mNG::3xMyc cell line. The cell line expressing KKT2 AS variant (KKT2^{M146G}) was engineered using CRISPR-Cas9 to endogenously tag the C-terminus of both alleles of KKT2 AS with mNeonGreen (mNG) fused to the 3xMyc epitope, resulting in the KKT2_AS::mNG::3xMyc cell line. A repair template containing a blasticidin resistance marker and linear DNA fragments for *in vivo* transcription of the single guide RNA was transfected into the parental line. Following an overnight recovery period, transfected cells were selected with blasticidin and subsequently cloned by serial dilution. (a) PCR amplification was performed to confirm the integration of the repair template at the correct locus in both alleles. The diagram on the right illustrates the PCR strategies and the expected DNA product sizes for each amplification. The clone highlighted in yellow was selected for subsequent experiments. (b) Growth curves of *L. mexicana* promastigotes were assessed for the parental T7/Cas9, KKT2 AS, KKT2::mNG::3xMyc, and KKT2_AS::mNG::3xMyc lines. Growth rates were calculated from the logarithmic phase of the growth curve (24 h) and are reported as mean ± SEM: T7/Cas9, 2.07 ± 0.128; KKT2_AS, 2.09 ± 0.091; KKT2::mNG::3xMyc, 2.058 ± 0.082, and KKT2_AS::mNG::3xMyc, 1.82 ± 0.059. Statistical comparisons between mutants and the parental T7/Cas9 line were performed using unpaired two-tailed Student's t-test, and no significant differences in growth rates were observed.
