# Supplementary material for: Chemical genetics reveals Leishmania KKT2 and CRK9 kinase activity is required for cell cycle progression
Source: PLoS Pathog. 2026 May 13;22(5):e1014194. doi: 10.1371/journal.ppat.1014194 (PMC13211308; doi:10.1371/journal.ppat.1014194)
Supplement: S18 Fig — (PDF) [file ppat.1014194.s022.pdf]

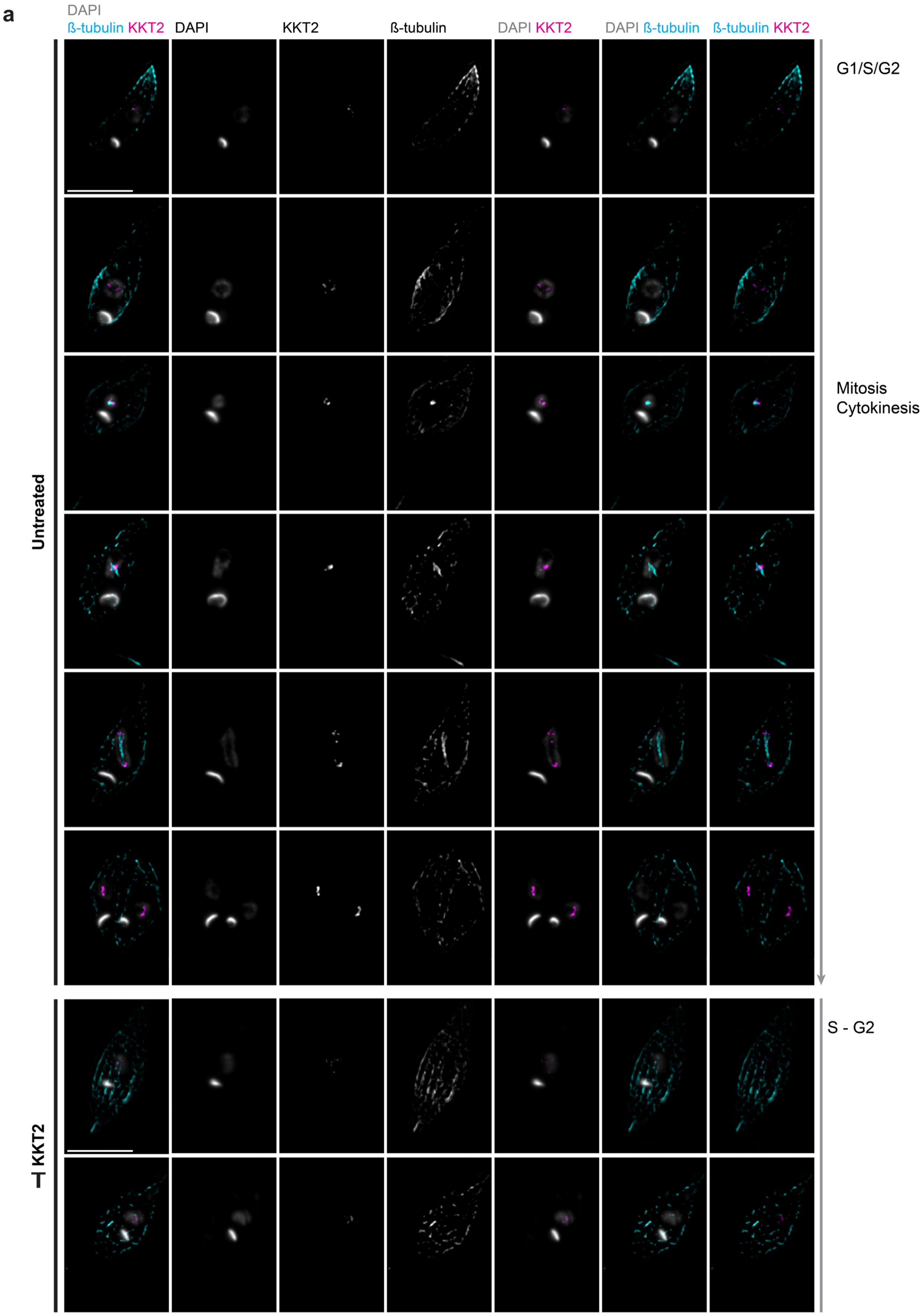

S18 Fig. Spatial and temporal distribution of *L. mexicana* KKT2 throughout the cell cycle.

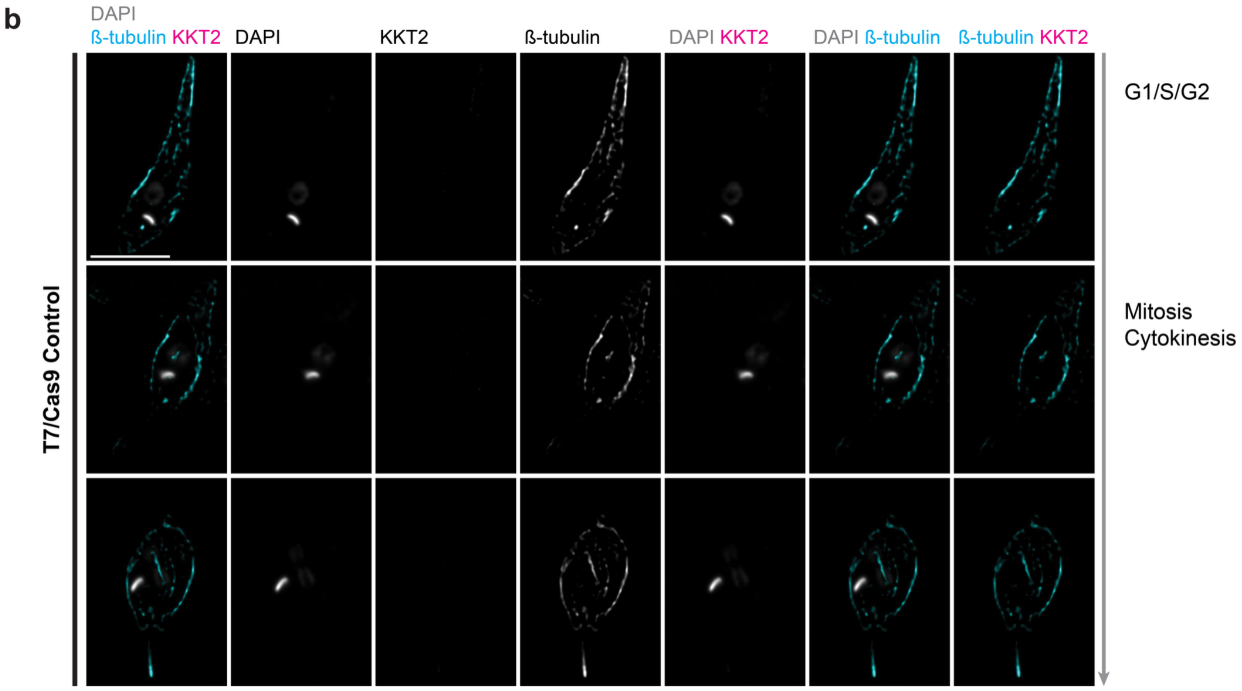

S18 Fig. Spatial and temporal distribution of *L. mexicana* KKT2 throughout the cell cycle. High-resolution fluorescence microscopy was performed on KKT2_AS::mNG::3xMyc promastigotes cultured in the presence or absence of 10 µM 1NM-PP1 for 6 hours (a) or on the untagged T7/Cas9 control line (b). Cells were stained with KMX-1 antibody to detect β-tubulin, anti-Myc antibody to visualize KKT2, and counterstained with DAPI for DNA visualization. Representative fluorescence micrographs show the localization of KKT2 and β-tubulin across different *Leishmania* cell cycle stages. The colours used for each marker are indicated to the top of the images. Scale bars, 5 µm.
