# Supplementary material for: Chemical genetics reveals Leishmania KKT2 and CRK9 kinase activity is required for cell cycle progression
Source: PLoS Pathog. 2026 May 13;22(5):e1014194. doi: 10.1371/journal.ppat.1014194 (PMC13211308; doi:10.1371/journal.ppat.1014194)
Supplement: S19 Fig — (PDF) [file ppat.1014194.s023.pdf]

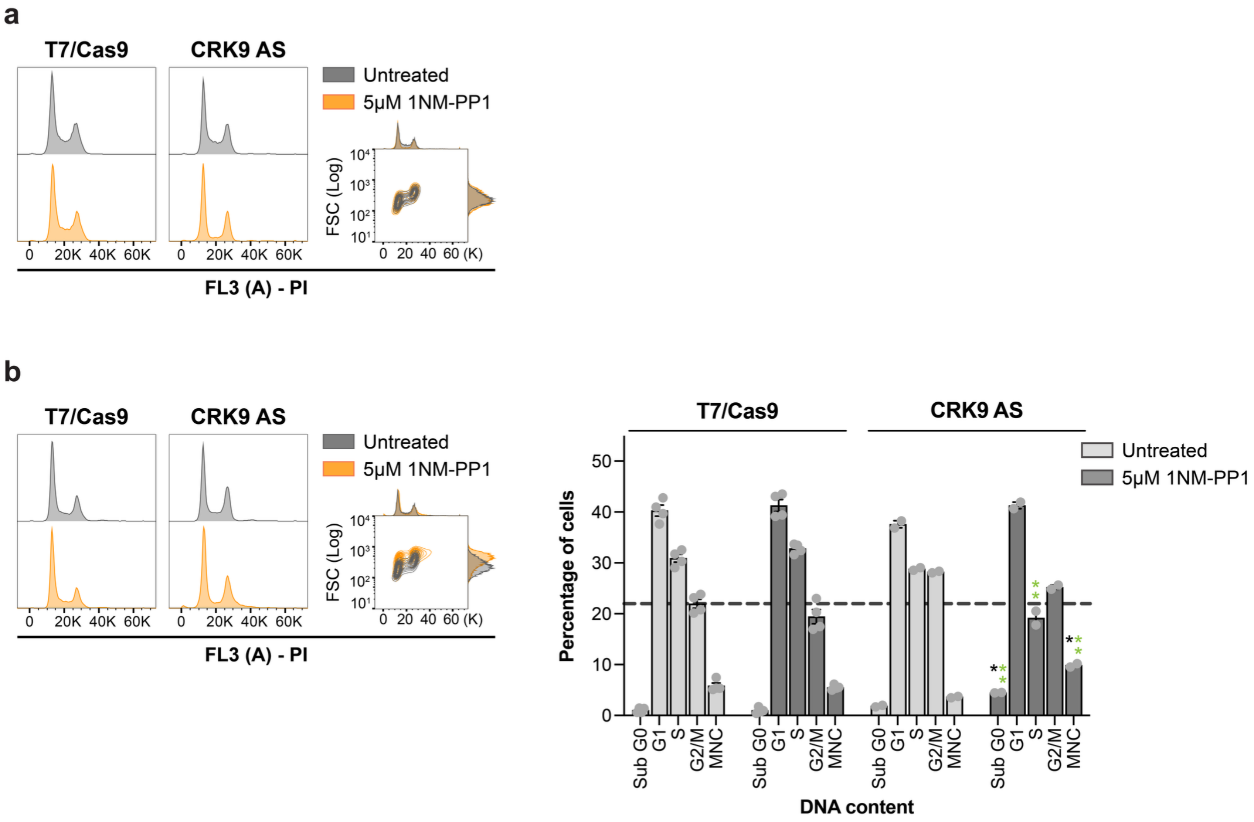

S19 Fig. Effect of CRK9 kinase activity inhibition on *Leishmania* cell cycle progression. The parental T7/Cas9 line and untreated parasites expressing CRK9 AS variant ($CRK9^{M501G}$) cultured under the same conditions were used as controls. Cell cycle analysis of cells stained with propidium iodide (PI) after 6 hours (a) or 24 hours (b) of treatment with 5 µM 1NM-PP1. Cell cycle phase quantification was performed using the Watson model algorithm in FlowJo v10.10.0. The left panel displays a representative cell cycle histogram, with adjacent histograms showing DNA content and forward scatter (FSC) were used to assess cell size across different cell cycle stages. The right panel presents the percentage of cells in each cell cycle phase. Two-tailed Student's t-tests were used to compare (i) treated *versus* untreated analog-sensitive populations (black), and (ii) wild-type versus analog-sensitive populations under treated conditions (green) (* $p < 0.05$; ** $p < 0.01$). Data are mean ± SEM of four biological replicates. G, gap phase; S, synthesis phase; M, mitosis phase; MNC, multinucleated cells; Sub-G0, cells with less DNA than typical G1-phase cells, indicative of DNA degradation.
