# Supplementary material for: Chemical genetics reveals Leishmania KKT2 and CRK9 kinase activity is required for cell cycle progression
Source: PLoS Pathog. 2026 May 13;22(5):e1014194. doi: 10.1371/journal.ppat.1014194 (PMC13211308; doi:10.1371/journal.ppat.1014194)
Supplement: S20 Fig — (PDF) [file ppat.1014194.s024.pdf]

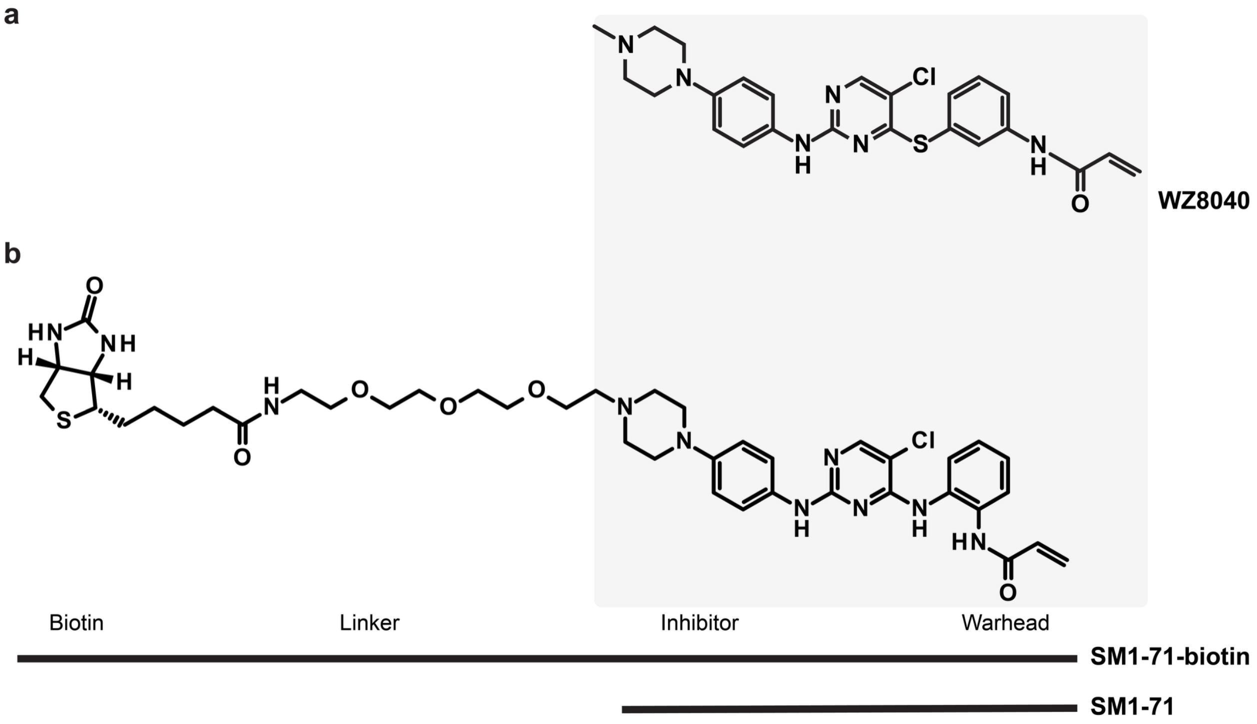

S20 Fig. WZ8040 and SM1-71 probes. (a) Chemical structure of WZ8040 kinase inhibitor. (b) Chemical structures of SM1-71 and its biotinylated analog.
