# Supplementary material for: Chemical genetics reveals Leishmania KKT2 and CRK9 kinase activity is required for cell cycle progression
Source: PLoS Pathog. 2026 May 13;22(5):e1014194. doi: 10.1371/journal.ppat.1014194 (PMC13211308; doi:10.1371/journal.ppat.1014194)
Supplement: S21 Fig — (PDF) [file ppat.1014194.s025.pdf]

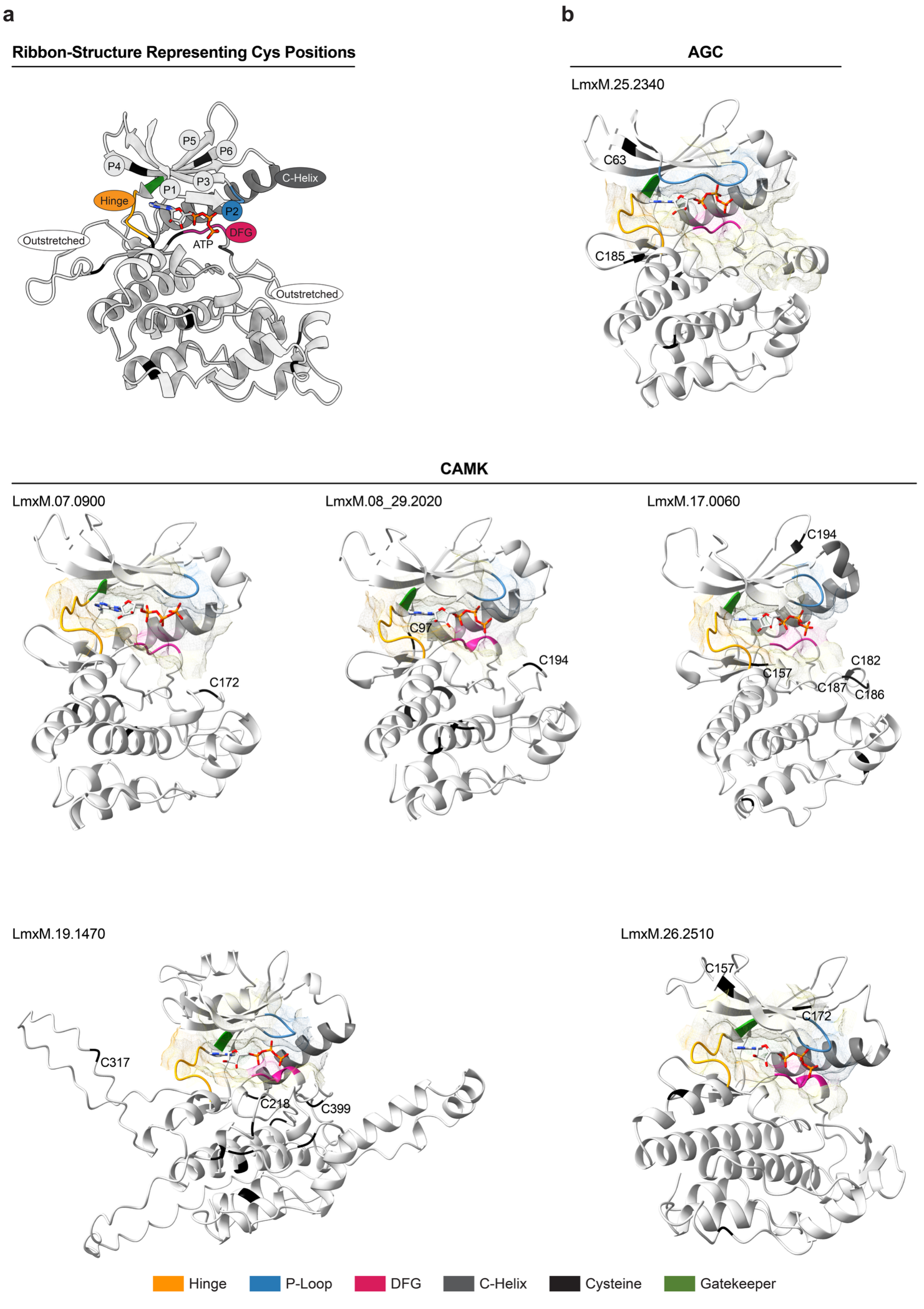

S21 Fig. Predicted structural models of kinase domains from *L. mexicana* protein kinases enriched by the multi-targeted acrylamide-modified probe, SM1-71-biotin.

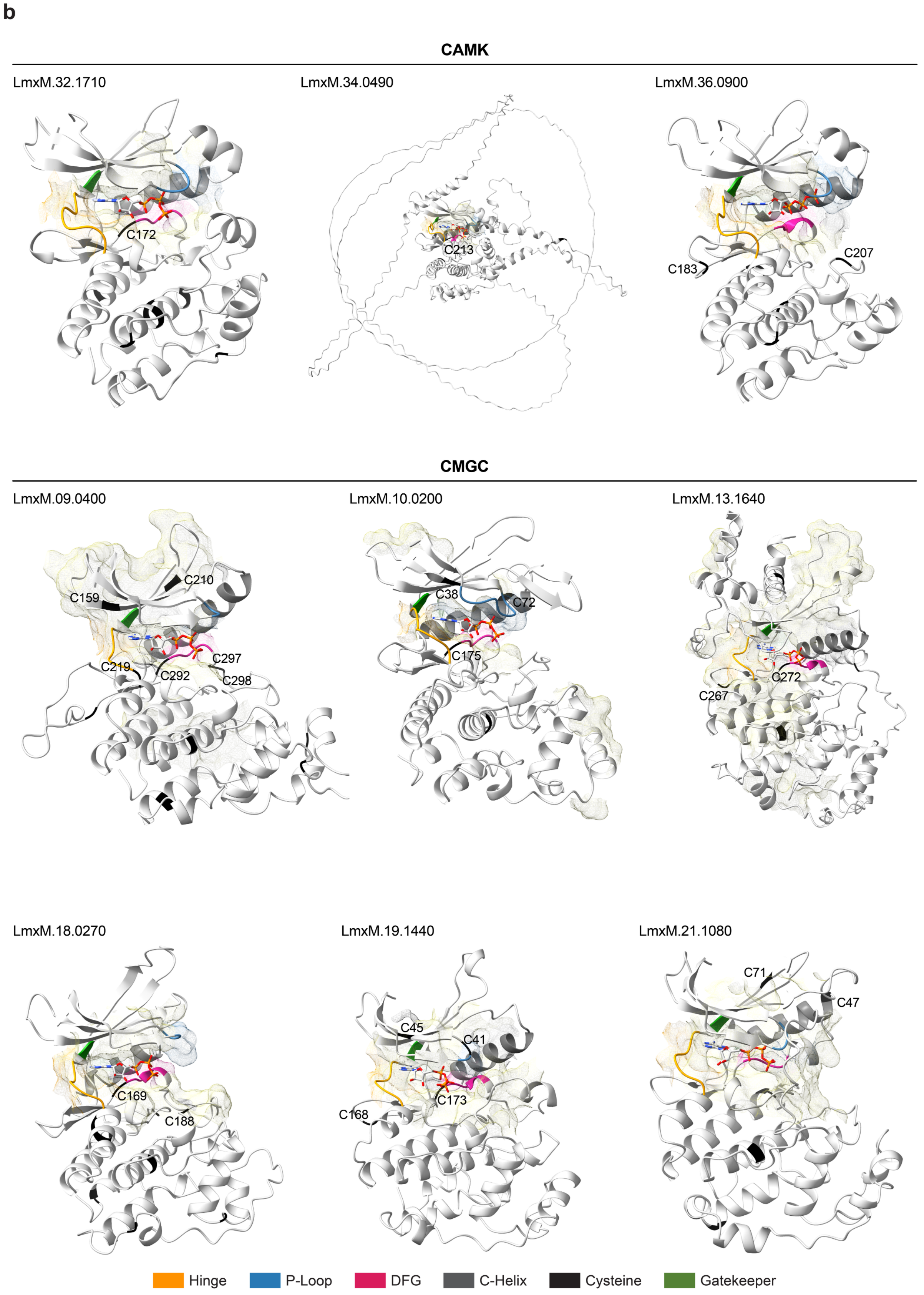

S21 Fig. Predicted structural models of kinase domains from *L. mexicana* protein kinases enriched by the multi-targeted acrylamide-modified probe, SM1-71-biotin.

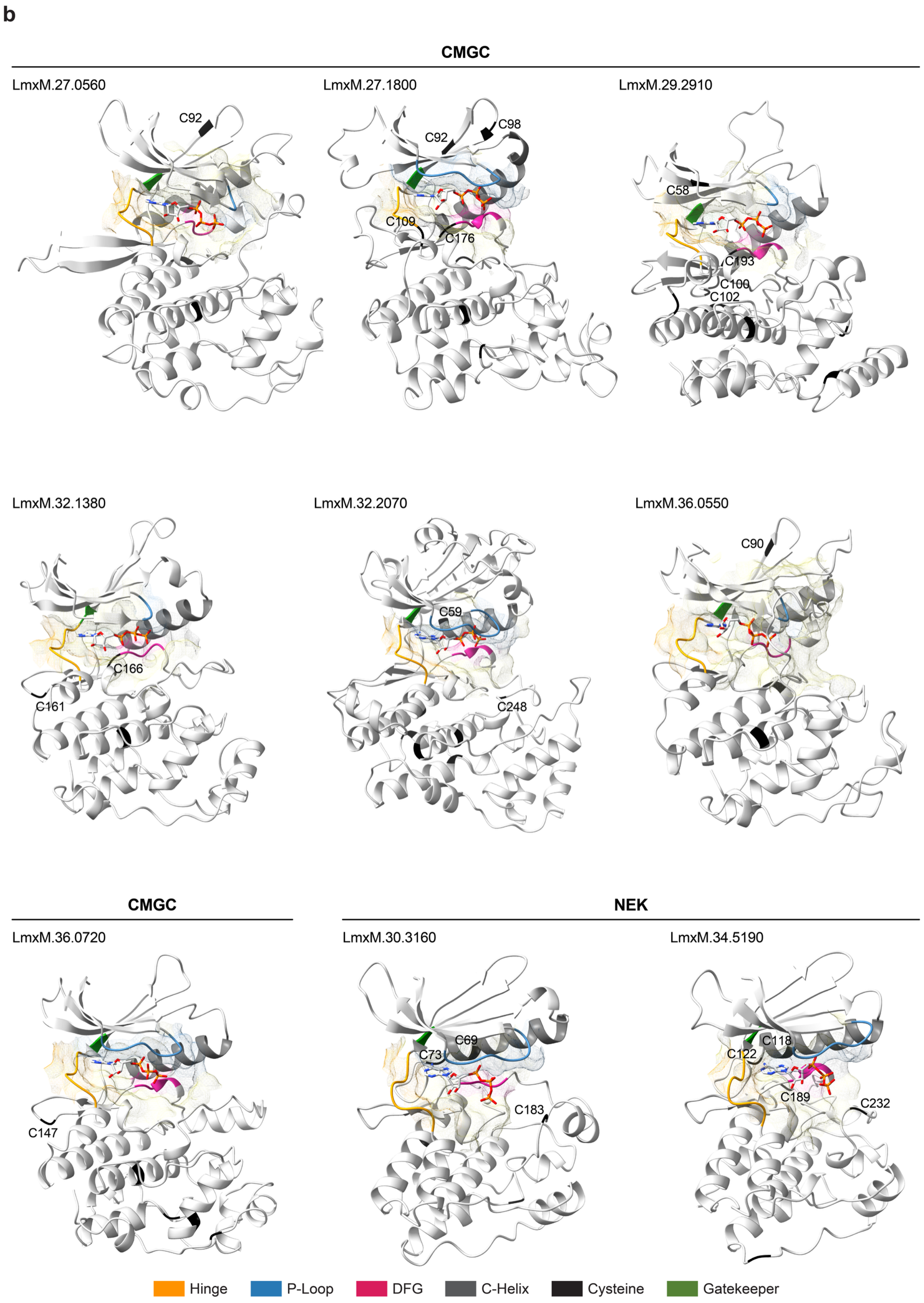

S21 Fig. Predicted structural models of kinase domains from *L. mexicana* protein kinases enriched by the multi-targeted acrylamide-modified probe, SM1-71-biotin.

b

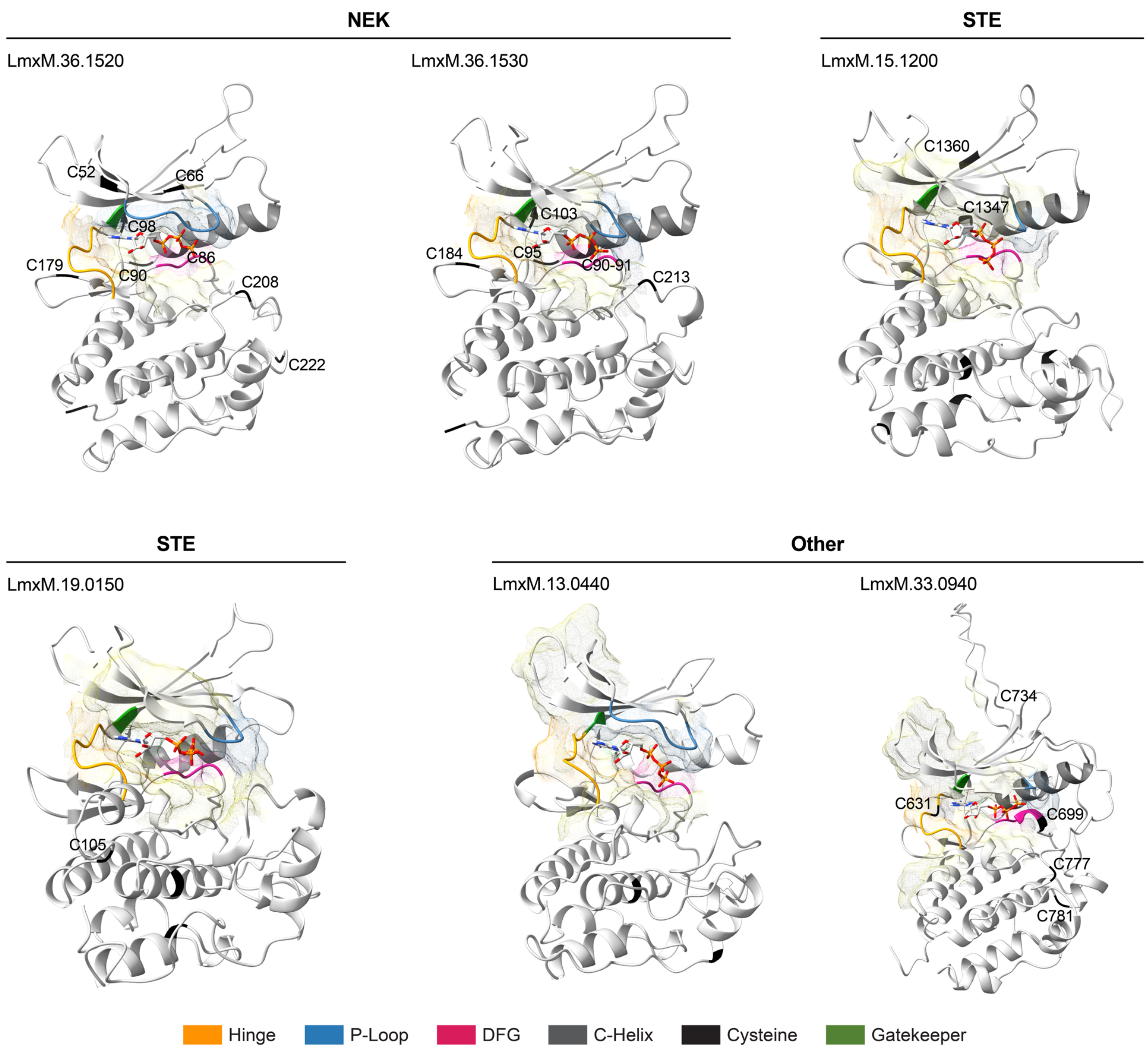

S21 Fig. Predicted structural models of kinase domains from *L. mexicana* protein kinases enriched by the multi-targeted acrylamide-modified probe, SM1-71-biotin. Structural models of the kinase domains were generated using AlphaFold 3 [1] and visualized with ChimeraX v1.9. The ATP ligand is displayed as a stick model, with heteroatom-based colouring. Residues located within 6 Å of the ATP-binding site are represented as a semitransparent surface overlay. AlphaFold confidence metrics, including the predicted template modelling score (pTM) and the interface predicted template modelling score (ipTM) for kinase-ATP interaction, are provided in Supplementary Data 3. (a) Ribbon diagram indicating the positions of cysteine residues within the kinase domain. (b) Predicted structural models of kinase domains from *L. mexicana* protein kinases identified as enriched by the multi-target acrylamide-based probe SM1-71-biotin.

References

1. Abramson J, Adler J, Dunger J, Evans R, Green T, Pritzel A, et al. Accurate structure prediction of biomolecular interactions with AlphaFold 3. Nature. 2024;630(8016):493-500. Epub 20240508. doi: 10.1038/s41586-024-07487-w. PubMed PMID: 38718835; PubMed Central PMCID: PMCPMC11168924.
