# Supplementary material for: Chemical genetics reveals Leishmania KKT2 and CRK9 kinase activity is required for cell cycle progression
Source: PLoS Pathog. 2026 May 13;22(5):e1014194. doi: 10.1371/journal.ppat.1014194 (PMC13211308; doi:10.1371/journal.ppat.1014194)
Supplement: S22 Fig — (PDF) [file ppat.1014194.s026.pdf]

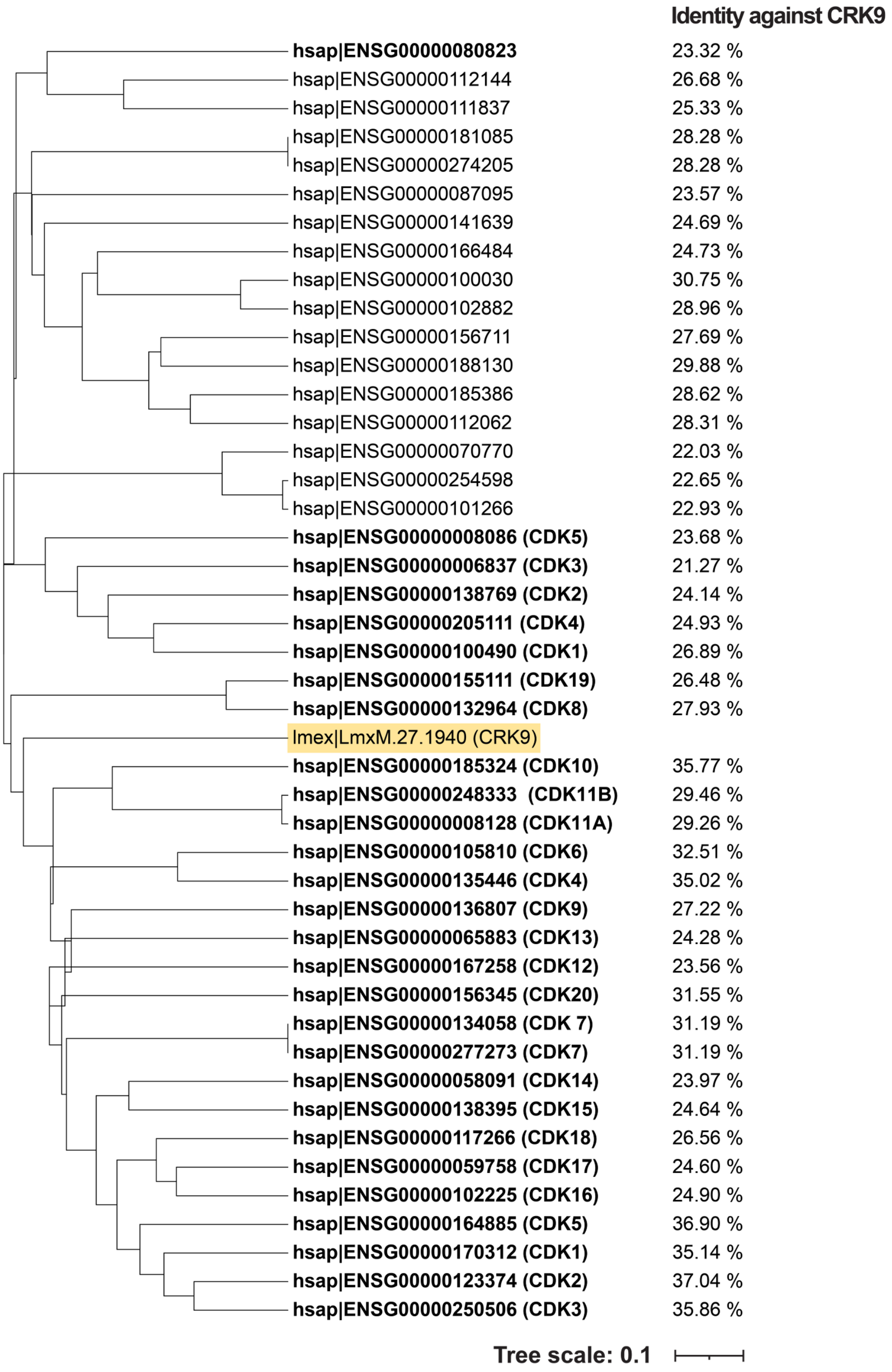

S22 Fig. Phylogenetic analysis of human CDKs with sequence similarity to *L. mexicana* CRK9. Phylogenetic tree visualization of *L. mexicana* CRK9 (LmxM.27.1940) and selected human CDKs, as identified using OrthoMCL BD (https://orthomcl.org/orthomcl/app). The *L. mexicana* CRK9 protein is highlighted in yellow, and human cyclin-dependent kinases (CDKs) are indicated in bold. Protein sequences were aligned using Clustal Omega (https://www.ebi.ac.uk/jdispatcher/msa/clustalo), and the percentage identity relative to *L. mexicana* CRK9 is shown on the right side of the tree.
