# Supplementary material for: Chemical genetics reveals Leishmania KKT2 and CRK9 kinase activity is required for cell cycle progression
Source: PLoS Pathog. 2026 May 13;22(5):e1014194. doi: 10.1371/journal.ppat.1014194 (PMC13211308; doi:10.1371/journal.ppat.1014194)
Supplement: Attachment — Submitted filename: Response to Reviewers - PlosPathogens.docx [file ppat.1014194.s031.docx]

PPATHOGENS-D-25-02309
Chemical genetics reveals Leishmania KKT2 and CRK9 kinase activity is required for cell cycle progression

PLOS Pathogens

**Please find below our response to the Reviewers**

**Reviewers' Comments:**

Reviewer's Responses to Questions

**MAJOR ISSUES: KEY EXPERIMENTS REQUIRED FOR ACCEPTANCE**
Please use this section to detail the key new experiments or modifications of existing experiments that should be absolutely required to validate study conclusions.

**Reviewer #1:**

1- The section, Leishmania kinome, a source of attractive drug targets is not really required for the investigation described in the title. Nevertheless, I include observations related to its content as they are important to rectify mistakes made earlier.

This analysis was conducted to identify protein kinases thought to be essential for amastigote survival that contain natural glycine or alanine gatekeeper residues. We did not identify any such kinases, however, we identified protein kinases harbouring a serine (LmxM.36.4250) or a threonine (LmxM.13.1640, LmxM.21.1650, LmxM.29.0370, and LmxM.29.2910) residue at this position, which have previously been reported to confer sensitivity to bumped kinase inhibitors. Since our analysis indicated that some of these kinases are required for amastigote survival in an *in vivo* mouse model, this has implications for applying AS kinase technology in *Leishmania* studies using mouse models. Therefore, we believe that this analysis should remain part of the manuscript, and we have incorporated it into the revised discussion.

1.1- Lines 169-172 Cytoplasmic localization in promastigotes does not necessarily mean the protein is also expressed in amastigotes and found in the same compartment.

This is true, therefore, we have removed the “Primary localization” column from Table 1, as well as the corresponding sentence in the Results section referring to the subcellular localization of these protein kinases.

1.2- There appears to be something wrong with MKK1 and MKK4 in the Baker et al paper Nat Commun 12, 1244 (2021), which seems to carry over into this manuscript. LmxM.08_29.2320 MKK1 is shown with a red dot in the kinase tree of figure 1 a and hence is labelled as “could not be deleted” by Baker et al using CRISPR-Cas9 technology. In figure 3 c (2c) in Baker et al a deletion of MKK1 in the presence of a trans gene is documented. Was that the cell line that was used in the pool with the null mutants of the other kinases in this manuscript? It remains unclear whether Baker et al could not generate a null mutant for MKK1 or whether a null mutant for MKK1 did not show a motility defect. MKK1 could be deleted in a previous publication, EUKARYOTIC CELL, 2003, 769–777 and the null mutant was found to be able to cause lesions in Balb/c mice and differentiate back into promastigotes. Moreover, MKK1 (LmxMKK) was shown to be absent in lesion-derived amastigotes of L. mexicana wild type using immunoblot analysis with an MKK1 specific antiserum.

For consistency, we used the data from Baker et al. to give us a long list of protein kinases predicted to be required for amastigote survival (Table 1). Large scales screens may contain inconsistencies and whilst an *MKK1* null mutant could not be generated using CRISPR in that study, *MKK*1 could be deleted using homologous recombination (Eukaryotic Cell paper). This finding (and associated phenotyping) is significantly more robust than the CRISPR study, so we have removed MKK1 from Table 1 and added a note to Supplementary Data 1.

1.3- For noting: In table 1, the Baker et al paper claims that LmxM.24.2320 MKK4 (LmxPK4) could not be deleted using CRISPR-Cas9 technology. However, this kinase was not labelled with a red dot in figure 1. Again, in a previous publication Molecular Microbiology (2005) 56(5), 1169–1182, null mutants for this kinase could be generated using homologous recombination. In case the authors decide to correct their previous publication, they should also check their references for correct numbering, occurrence and fit.

We thank the reviewer for these points. We have added a note in Supplementary Data 1 of the present manuscript indicating that MKK4 was successfully deleted from the *L. mexicana* genome in a previous study. This information will help guide future selection of protein kinases for drug target validation.

1.4- Table 1 of the manuscript under review shows PK4 as LmxM.21.1650. Please check whether this should be LmxM.24.2320 or a different name.

LmxM.21.1650 is named PK4 in our previous systematic functional analysis of *Leishmania* protein kinases (Baker, N. et al., Nature Communications 12:1244, 2021), whereas the gene LmxM.24.2320 is named MKK4.

1.5- Information on null mutants of MPK5 and MPK12 has been published in the International Journal for Parasitology 37 (2007) 1053–1062. MPK5 null mutants persist in infected mice without lesion development and MPK12 null mutants are as infective as L. mexicana wild type. Additionally, LmxMPK7 genomic null mutants carrying an episomal copy of the kinase gene lost this copy when cultured as promastigotes in the absence of selective pressure or when used to infect Balb/c mice (Bleicher N, PhD thesis, Hamburg, <https://ediss.sub.uni-hamburg.de/handle/ediss/8014>). The virulence of LmxMPK7 null mutants was significantly reduced causing delayed lesion development or no lesions at all. However, using null mutants obtained from mouse lesions led to normal lesion development in a subsequent infection without delay suggesting compensation. This suggests that MPK7 might not be the best drug target.

We thank the reviewer for raising these important points and agree that all available evidence should be considered when prioritising potential drug targets. In the revised manuscript, we have included discussion about the targets MPK4, MPK5, MPK7 and AEK1. In contrast, we do not further discuss MPK12, as it was not highlighted as a kinase containing an accessible reactive cysteine.

1.6- I assume the final time point in the barcode experiment is 6 weeks post infection. Please, explain why a 50% decrease is sufficient to assume that the respective kinase is essential for survival of the amastigotes. Would you not expect to see a complete loss of those mutants having lost a kinase that is essential?

“A protein kinase was classified as required for amastigote survival if the corresponding mutant cell line exhibited the following trajectory in barcode representation: (i) a significant ≥2-fold reduction in barcode abundance at the first post infection time point, followed by an additional decrease of ≥30% at the second time point (meaning ≥0.5 at first time point followed by ≥0.35 at second); or (ii) no significant change in the first time point, but a significant ≥50% reduction at the final time point (meaning ≥0.5 at last time point, but still the previous time point being higher) (Supplementary Fig 1).” Might not be sufficiently stringent.

Yes, six weeks represents the final time point in the barcode experiment. In the library-based barcode experiment, a specific *Leishmania* cell line can be influenced positively, negatively, or not by the host. In this context, we believe that applying two cutoff criteria, a 2-fold reduction in abundance and a statistical test (p-value < 0.05), is a reasonable approach to identify loss of fitness for amastigotes in the host. This may not be particularly stringent, but that is why the chemical genetics approach for target validation described in the is paper is important to add value to genetic validation

Also, replace “≥2-fold reduction” with “≥50% reduction” for consistency.

Replaced.

1.7- It is not clear how the above criteria were used to analyse the data presented in supplementary data 1. CLK1, CLK2, KKT2, and KKT3 are shown as not present in the library, hence, no ratios can be determined but they are still labelled as “Required” in macrophages. In addition, why are LmxM.08_29.2020, LmxM.36.0720, and LmxM.34.4620 not required in macrophages? They seem to fulfil the criteria. Likewise, to name a few others, for LmxM.19.1470, LmxM.22.0810, and LmxM.04.1210 it is not clear why they are “Required” based on the data for the situation in macrophages. It looks like that the trajectory from FP3 to FP6 for LmxM.04.1210 is pointing upwards rather than down. I might misinterpret the numbers. To avoid this from happening a better explanation is needed.

Thank you for drawing our attention to this point. We apologize for the oversight and have now added to Supplementary Data 1 our recent references demonstrating that CLK1/CLK2 are required in the promastigote stage. Using the Novartis covalent inhibitor AB1, we have also observed that CLK1/CLK2 are required for amastigote survival. However, as this study has not yet been approved for publication, we have removed the CLK1/CLK2 amastigote-stage data from the current manuscript and have made the necessary corrections in the text.

Based on our previous study (Baker, N. et al., Nature Communications 12:1244, 2021), KKT2 and KKT3 were reported in Supplementary Data 1 as “Required” for promastigote survival. For *in vitro* infected macrophages *and in vivo* mouse footpad infections, both genes were classified as “Not known,”. Consequently, the criteria described above were not applied to assess KKT2 and KKT3, and the manuscript does not claim that these criteria were used for these kinases (KKT2 and KKT3 are not included in Table 1 of this manuscript).

In the current study, after generating the KKT2 analog-sensitive mutant, we were able to confirm that KKT2 is also required for amastigote survival. For KKT3, however, further investigation is needed to determine whether it is required for amastigote survival.

We have revised the analysis to identify protein kinases required for amastigote survival and to address the specific points raised by the reviewer:

- LmxM.08_29.2020: The p-value for the comparison between 12 h and 72 h is not statistically significant. According to our criteria, both a defined fold-change threshold and statistical significance must be met; therefore, this kinase was not classified as required.
- LmxM.36.0720 & LmxM.34.4620: When the first time point does not meet the classification criteria, we require a minimum 50% reduction at the second time point. Neither LmxM.36.0720 (72 h/12 h fold-change = 0.54) nor LmxM.34.4620 (72 h/12 h fold-change = 0.52) satisfied this threshold.
- LmxM.19.1470, LmxM.22.0810, and LmxM.04.1210: Based on our criteria, these kinases were classified as “Dispensable” in the *in vitro* infected macrophage (inMAC) model and as “Required” in the *in vivo* mouse footpad model. Indeed the trajectory of LmxM.04.1210 in the inMAC model trends upward rather than downward, but it was not classified as required in the inMAC model.

1.8- To conclude, this section needs to be corrected and much improved or removed in its entirety, as the analysis does not lead to the selection of the four mentioned kinases, nor CRK9.
The section on checking whether Leishmania might be suitable for testing using bumped kinase inhibitors does not warrant that much space and could possibly reduced to a few sentences.

This analysis was performed to identify protein kinases potentially required for amastigote survival, which could impact the results of our susceptibility assays if they were naturally sensitive to bumped kinase inhibitors. In addition, based on the positive feedback from Reviewer 2 regarding this section, we believe that this analysis should be retained in the manuscript.

2- Line 286, check the Jones NG publication. There is no CRK9 mentioned in the cited paper. CRK9 AS data for promastigotes should be included.

CRK9 AS data are presented in the cited paper’s Supplementary Information (Supplementary Figure 10), which we have reproduced below:

Jones NG, Geoghegan V, Moore G, Carnielli JBT, Newling K, Calderon F, et al. Bromodomain factor 5 is an essential regulator of transcription in *Leishmania*. Nat Commun. 2022;13(1):4071. Epub 20220713. doi: 10.1038/s41467-022-31742-1. PubMed PMID: 35831302; PubMed Central PMCID: PMCPMC9279504.

3- In line 323 onwards, the authors argue that KKT3 is required in Leishmania but not for its kinase activity on the basis that the AS mutant version does not show an effect in cells treated with the inhibitor. There is no evidence provided that KKT3 AS is actually sensitive to the inhibitor. This approach does not work for all kinases. Unfortunately, KKT3 is a big molecule but recombinant expression of the kinase domain (amino acids 1-337) might be possible for the wild type and AS mutants followed by enzymatic assays to assess whether the AS mutant can be inhibited. The authors mention further experiments are required later in line 545 onwards.

Indeed, we attempted to obtain recombinant KKT3 by generating 17 different wild-type constructs and 2 AS mutants (gatekeeper Met→Ala and Met→Gly) in *E. coli*, but none yielded soluble protein under a range of expression conditions.

To further investigate the requirement of KKT3 kinase activity, as requested by the reviewers we attempted to generate catalytically inactive (kinase-dead) KKT3 mutant using precision editing, either through mutation of the three key catalytic residues (K64, D156, and D174) to alanine, or by deleting residues 43 – 327, which encompass nearly the entire kinase domain (residues 36 – 335). However, to our surprise, it was not possible to recover kinase-dead mutations, nor a null mutant of the kinase domain. We only managed to recover clones with the catalytic aspartate D156 mutated, and with one allele of KKT3 kinase domain deleted. We observed that the KKT3^D156A^ mutant displayed a significantly reduced growth rate during the logarithmic phase, whereas parasites carrying a heterozygous deletion of the KKT3 kinase domain exhibited a non-significant trend towards reduced growth. Altogether, these findings suggest that KKT3 kinase activity is likely essential for parasite survival and that gatekeeper substitution alone does not sensitize KKT3 to inhibition by 1NM-PP1. These results have been incorporated, and the manuscript has been revised accordingly.

4- Figure 4 d requires a wild type control for the myc tag or better even an immunoblot showing that the anti-myc antibody used here only recognises the tagged KKT2.

We thank the reviewer for drawing our attention to the missing figure. The corresponding control images have now been added to Supplementary Fig. 18 in the revised manuscript.

**Reviewer #3:**

1- The authors should evaluate recombinant versions of the “bumped” kinases in an in-vitro kinase assay to verify their direct inhibition by 1NM-PP1 and 1NA-PP1. These experiments would clarify the mechanistic differences between the two analogues and rule out the possibility that the absence of a phenotype for KKT3 is simply due to ineffective inhibition of its catalytic activity. Demonstrating that KKT3 kinase activity can be blocked without affecting cell viability would provide strong support for the authors claim that “KKT3, but not its kinase activity, is essential for promastigote viability.”

We agree with the reviewer’s point. However, despite extensive efforts, we were unable to generate recombinant AS versions of KKT2, KKT3, and CRK9 in quantities and quality suitable for biochemical characterization. For KKT2, we cloned and expressed a total of 20 wild-type (WT) constructs and 2 AS mutants (gatekeeper Met→Ala and Met→Gly) in *E. coli*, as well as 3 WT constructs in Sf9 insect cells. Although WT KKT2 could be produced in both expression systems, expression of the AS mutants in *E. coli* resulted in extremely low yields. For KKT3, we generated 17 WT constructs and 2 AS mutants (gatekeeper Met→Ala and Met→Gly) in *E. coli*, but none yielded soluble protein under a range of expression conditions. Similarly, for CRK9, 17 WT constructs and 2 AS mutants (gatekeeper Met→Ala and Met→Gly) were successfully cloned, yet all recombinant proteins expressed in *E. coli* were insoluble. Collectively, these results indicate that these proteins are recalcitrant to recombinant production in *E. coli*, precluding biochemical characterization. We have therefore relied on cellular and genetic assays to assess the functional consequences of kinase inhibition.

Please also refer to our response to Major point 3 raised by Reviewer 1, in which we explain how we addressed the requirement of KKT3 kinase activity for parasite survival.

2- For the untreated control, the infection rate (percentage of infected macrophages) and the parasite burden (number of parasites per 100 macrophages) measured at different time points up to 96 hours (every 24h) for the parental strain and the strains containing the bumped kinases should be presented. This time-course data will allow a clear assessment of parasite survival, differentiation and multiplication in the absence of any treatment and determine whether the kinetic of infection is comparable between the parental strain and the strains containing the bumped kinases.

We analysed the percentage of infected macrophages at the end of the intracellular amastigote susceptibility assay under untreated conditions and compared the analog-sensitive kinase lines with the parental T7/Cas9 control. The comparable levels of infected macrophages indicate that the gatekeeper mutation is unlikely to have impaired parasite infectivity and/or proliferative fitness in the analog-sensitive lines. These results are presented in panel d of Supplementary Fig. 9 in the revised manuscript.

3- The authors should investigate whether the tested compounds have any activity on mouse macrophage proteins, particularly host kinases that might be naturally sensitive, to determine if off-target effects on the host cells could contribute to the observed differences in IC₅₀ values between promastigotes and amastigotes.

We assessed the cytotoxicity of the bumped kinase inhibitor 1NM-PP1 in uninfected bone marrow-derived macrophages (BMDM) using a resazurin-based assay. Significant cytotoxicity was observed only at concentrations ≥40 µM, corresponding to the highest concentration tested, with a 48.6% reduction in cellular metabolic activity. These results have been added to the revised manuscript and are presented in Supplementary Fig 9e.

4- The authors identified several kinases that contains targetable cysteines (Table 3). They should validate their results by selecting at least one kinase and perform a target engagement assay, such as cellular thermal shift assay (CETSA), with SM1-71 to demonstrate covalent inhibition.

We have carried out a mass spectrometry-based target engagement assay with recombinant CLK1 and SM1-71 (Fig 8 of the revised manuscript), demonstrating covalent binding. Additionally, we have previously demonstrated target engagement of CLK1 and the EGFR inhibitor WZ8040, using a bioluminescence resonance energy transfer (BRET)-based target engagement assay in live cells (Catta-Preta et al., 2025). WZ8040 is structurally very similar to SM1-71 (Supplementary Fig 20, and details included in the results and discussion of the revised manuscript)

**MINOR ISSUES: EDITORIAL AND DATA PRESENTATION MODIFICATIONS**
Please use this section for editorial suggestions as well as relatively minor modifications of existing data that would enhance clarity.

**Reviewer #1:**

1- Line 227 should be "related trypanosomatid". Delete “As” at the beginning of the sentence in line 400.

Thank you for pointing this out. Both points have now been corrected in the revised manuscript.

**Reviewer #2:** The authors might want to consider the points listed below.

1- Line 61: parasites (plural)

Thank you for pointing this out. We have corrected in the revised manuscript.

2- Line 62: “the mechanisms of action of most antileishmanial drugs remain incompletely understood.” I would suggest mentioning the mechanisms of resistance here, as the sentence later mentions drug resistance surveillance strategies; mechanisms of action and of resistance are often uncoupled (e.g efflux transporters). Suggestion: the mechanisms of action of most antileishmanial drugs, and the mechanism underlying resistance to these compounds, remain incompletely understood.

Suggestion accepted. The manuscript has been edited to incorporate the reviewer’s recommendation.

3- Line 118 ff: The idea of implementing a gatekeeper-based chemical genetics analysis for protein kinases of parasites has been proposed two decades ago (PMID: 12377287; one of the authors is the senior author of the present manuscript. It would be appropriate to cite this paper at the start of the paragraph in line 118.

We have included this reference.

4- Line 136 ff: In a similar vein, the targeting of Cysteine residues in PKs has also been explored with success in Plasmodium, another major protistan parasites, and, interestingly, in a PK of the CLK family (e.g. PMID: 39441986). It would be relevant to briefly mention this in the introduction, may at line 137, after citing Ref [6].

We have included this reference.

5- Line 199 ff: “Of the 193 predicted ePKs previously annotated in the L. mexicana kinome [14], 10 are classified as pseudokinases as they lack all three of the conserved catalytic residues (K D D) required for kinase activity (Fig 1a). Given their lack of enzymatic function and demonstrated non-essentiality, pseudokinases were not analysed for gatekeeper residue identity.” There has been debate about the total inactivity of pseudokinases (e.g. PMID: 20421461). How onerous would it be to check the gatekeeper of these 10 sequences labelled as pseudokinases? It would not hurt to include them.

We are aware of the ongoing debate regarding the potential of pseudokinases to have kinase activity. However, is sometimes difficult to identify their gatekeeper residue with confidence due to the divergent nature of their sequence and structure. As all ten pseudokinases were classified as “dispensable” for parasite survival in our analysis, identifying their gatekeeper residues would not provide information directly relevant to the scope or conclusions of this manuscript.

6- The BioID interactomics study performed for CRK9 yielded interesting results; was this also considered for KKT2?

Yes, we have used KKT2 AS as a spatial control for another protein kinase (MSK, Mitotic Spindle Kinase; bioRxiv 2025:2025.09.19.677349; doi: 10.1101/2025.09.19.677349). In that study, we identified proteins and phosphosites proximal to KKT2. However, under the inhibition conditions applied in that experiment, we did not observe significant differences in protein or phosphosite abundance between active and inhibited KKT2. We are currently optimizing the inhibition time to improve detection sensitivity.

7- Line 506ff: “This finding stands in contrast to several apicomplexan parasites, including Toxoplasma gondii [50], Cryptosporidium parvum [51], and Neospora caninum [52], where glycine residues are found at the gatekeeper position in calcium dependent protein kinase 1 (CDPK1), thereby enabling selective inhibition by bumped kinase inhibitors and supporting their development in a targeted therapeutic approach.” It may be interesting to briefly mention that phenotypic/functional studies could indeed be implemented for some of the kinases with natural small gatekeepers, by generating mutant with a large gatekeeper, and comparatively evaluating the biochemical response to BKIs (to which the mutant are insensitive). See for example the papers on Plasmodium PKG and the T618Q gatekeeper substitution (e.g PMID PMID: 19915077; 38372781).

Thank you for pointing this out. We have revised the discussion to incorporate the reviewer’s suggestion.

8- Line 526. “We were unable to generate CLK1/CLK2 AS mutants, probably because the mutation is not tolerated by the kinase”. It has been shown that the gatekeeper Gly or Ala mutation can severely impair kinase activity. In many cases, this can be rescued by introducing a compensatory mutation, that restores activity and maintains susceptibility to BKI (see PMID: 30801966 for a review). This may deserve a comment in the discussion as a possible way forward to assess the function of CLK1/2 (see my comment above about the use of recombinant kinases to test the effect of the Gatekeeper mutations --this could ascertain rescue by the secondary mutation, CLK1/2 are shown to be rendered inactive by the gatekeeper mutation).

Thank you for pointing this out. We have revised the discussion to incorporate the reviewer’s suggestion.

We successfully produced recombinant wild-type (WT) CLK1 as well as its analog-sensitive (AS) versions bearing gatekeeper mutations (Met to Ala or Gly). Next, we attempted to establish an enzymatic assay for the CLK1-AS mutants. Using the same peptide substrates that were effective for the WT kinase, no detectable kinase activity was observed for either AS mutants. Consequently, it was not possible to assess their inhibition by 1NM-PP1. In addition, we employed intact mass spectrometry to evaluate *in vitro* auto-phosphorylation of the purified WT and AS CLK1 proteins. Upon incubation with ATP, the WT kinase exhibited three distinct phosphorylation states, consistent with robust auto-phosphorylation activity. In contrast, no phosphorylation was detected for the Gly gatekeeper mutant, while the Ala mutant displayed a single phosphorylation state at very low abundance. These results indicate that the gatekeeper mutations affect *in vitro* auto-phosphorylation activity. Besides, differential scanning fluorimetry revealed distinct thermal unfolding profiles for recombinant CLK1 compared with the analog-sensitive variants, consistent with altered protein stability upon gatekeeper substitution. Taken together, these findings with purified proteins suggest that the lack of success to generate CLK1/CLK2 AS mutants in *Leishmania* parasites is likely due to a substantial impairment of kinase activity caused by the gatekeeper mutations.

These results have been incorporated into the revised manuscript.

9- Lines 539 ff: It is interesting that the KKT3 AS mutation did not have any effect on the viability of parasite upon BKI treatment, but that the gene cannot be deleted (or knocked-down) entirely. It would be of interest to briefly discuss if there are other recognisable domains in addition to the kinase domain (which can be deleted) on the KKT3 polypeptide, whose deletion would be lethal.

Please also refer to our response to Major point 3 raised by Reviewer 1, in which we explain how we addressed the requirement of KKT3 kinase activity for parasite survival. We have also included in the revised manuscript a protein sequence and structural analyses that revealed that *L. mexicana* KKT3, similarly to *T. brucei* KKT3, harbours a centromere localization domain and a divergent polo box domain (Supplementary Fig 15). However, further investigation is required to assess the contribution of these additional KKT3 domains to *Leishmania* parasite survival.

Based on our additional data, we have revised the Result and Discussion sections related to KKT3 to incorporate these findings.

10- Line 543: “Here, we cannot exclude the possibility that the gatekeeper residue substitution in AS-KKT3 failed to sufficiently expand the ATP-binding pocket to permit effective binding of bumped kinase inhibitors”. It might indeed be the case that BKI cannot access the catalytic pocket even in the gatekeeper mutant. Secondary mutations have been in some case been shown to sensitise the mutated kinase to BKI inhibition --this could also deserve a comment (see the same paper as mentioned in the comment above, PMID: 30801966).

Thank you for pointing this out. We have taken the reviewer’s comment into consideration when revising the text for KKT3.

11- Overall comment on the two aspects of the manuscript. The two aspects of the study (1: Validation of PKs by chemical genetics and 2: search for cysteine-containing kinase domains) seem a bit disconnected, and do not support each other in terms of data. The authors might want to write a separate paper focussing on the cysteine-targeting story. Alternatively, an additional paragraph in the discussion outlining a similar AS chemical genetic approach for MPK4, MPK5, MPK7, and AEK1, that harbour reactive cysteine residues amenable to covalent inhibition, may improve the general self-coherence of the manuscript. One (maybe wild) suggestion: could it be that CLK1/2 were refractory to the AS mutation be linked to the presence of a cysteine in the catalytic domain? It may be worth it investigating the literature (e.g. the paper cited above, PMID: 30801966) to determine if cysteine-containing PKs are over-represented in the subset of PKs that required a secondary mutation to implement the AS gatekeeper chemical genetics approach.

Thank you for pointing this out. We have revised the Discussion to suggest validation of the highlighted protein kinases using our analog-sensitive kinase chemical genetics platform.

We examined the position of cysteine residues in *L. mexicana* CLK1. However, in contrast to human G protein-coupled receptor kinase 2 (GRK2) described in the referenced study, CLK1 contains an isoleucine rather than a cysteine immediately following the catalytic lysine. Based on this comparison, it is more likely that the leucine present in the atypical DLG motif contributes to the loss of kinase activity observed following gatekeeper mutation. This information has been incorporated into the revised Discussion.

12- Overall comment on the use of recombinant kinases: in line with previous comment, and in relation to the discussion about fitness of the parasites carrying an AS mutation (line 310), it would be nice to measure, using recombinant kinases, (i) the enzymatic activity parameters of the mutant kinases (in relation to the WTs), and (ii) the shift in IC50 for the BKIs. This is not a requirement, as the controls used throughout the study (effect of the BKIs on wild-type parasites and mock-treatment of the AS mutant) are sufficient to assign the phenotypic effects to the PK of interest, KKT3 and CRK9. However, with respect to CLK1/CLK2 (for which AS mutants could not be obtained), it might provide an answer to the question of whether this lack of success is caused by an impairment of kinase activity because of the mutation (see also above my comment on Line 526)

We undertook extensive efforts to generate recombinant wild-type (WT) and analog-sensitive (AS) variants of KKT2, KKT3, and CRK9. However, we were unable to generate recombinant AS versions of KKT2, KKT3, and CRK9 in quantities and quality suitable for biochemical characterization. Please also refer to our response to Major point 1 raised by Reviewer 3, in which we explain our attempts to obtain these recombinant proteins.

For CLK1 a total of 16 WT constructs and 2 AS mutants (gatekeeper Met→Ala and Met→Gly) were successfully cloned, and small-scale test expressions in *E. coli* showed that both WT and mutant proteins could be produced in soluble form. As soluble preparations of both wild-type CLK1 and its AS variants were obtained, we proceeded with biochemical characterization. These analyses showed that the gatekeeper mutation resulted in a loss of kinase activity. Please also refer to Minor Point 8 for additional details on the biochemical characterization of the CLK1 AS variants.

These data have been incorporated into the revised manuscript.

**Reviewer #3:**

1- The authors should provide the list of the 43 essential protein kinases they previously identified, as a supplementary table, thereby making the information more readily accessible. They should also mention that KKT2 and KKT3 were selected from the 43 essential protein list, while CLK1/2 were selected from Table 1, protein kinases only essential in amastigotes.

As suggested, we have now included the list of the 43 essential protein kinases previously identified in the promastigote stage, in Supplementary Table 1. In addition, we have corrected the classification of CLK1/CLK2 in promastigote stage to “required.” Our previous data demonstrate that CLK1 and CLK2 exhibit biological redundancy and share 100% sequence identity within their kinase domains. We have validated their essentiality for parasite survival using the covalent inhibitor AB1 and the compound WZ8040. As these small molecules target both kinases simultaneously, selective inhibition of only one is not feasible, and dual inhibition leads to cell death. For this reason, we classify CLK1/CLK2 as “required.” A corresponding note has been added to Supplementary Table 1 and Supplementary Data 1 to clarify this point. Please also refer to our response to Major point 1.7 raised by Reviewer 1, in which we explain about CLK1/2 requirement in promastigote and amastigote.

2- The authors should provide, as supplementary material, alphafold models of CLK1/2 and KKT3 both with and without the glycine/alanine residues. This will allow evaluation of whether the resulting conformational changes might explain the lack of editing tolerance for CLK1/2 or the loss of drug efficacy for KKT3.

Although AlphaFold is a highly accurate deep-learning method for predicting protein three-dimensional structures from sequence, it was not designed to predict the functional or stability consequences of single amino acid substitutions.

Instead, we used wild-type kinase models generated by AlphaFold3 in Missense3D to perform a predictive structural analysis comparing ATP-binding pocket expansion across all kinases in this study (KKT2, KKT3, CRK9, and CLK1). In addition (for curiosity), we explored the AlphaFold3 ipTM (interface predicted Template Modelling) metric, which quantifies confidence in predicted protein-ligand or protein-protein interfaces (with scores >0.8 indicating high-confidence interactions), to assess whether the gatekeeper mutation affected ATP binding. These results are presented in the table below and indicate that there is no direct correlation between predicted ATP pocket expansion or ATP-binding ipTM scores and our experimental observations. Specifically, although CLK1 was predicted to have one of the smallest ATP-binding pocket expansion following the gatekeeper mutation, the analog-sensitive CLK1 variant was experimentally shown to bind the bulky kinase inhibitor 1NM-PP1. Differential scanning fluorimetry (DSF) revealed thermal shifts (ΔTm) of 10.4°C and 3.9°C for CLK1^M213A^ and CLK1^M213G^, respectively, in the presence of 1NM-PP1 (unpublished data). Given this inconsistency between structural predictions and experimental observations, we chose not to include the Missense3D analysis in the revised manuscript.

|  | KKT2^M146^ | KKT3^M110^ | CRK9^M501^ | CLK1^M213^ |
| --- | --- | --- | --- | --- |
| ATP pocket expansion: M → G | 109.1 Å^3^ | 99.8 Å^3^ | 115.8 Å^3^ | 94.4 Å^3^ |
| ATP pocket expansion: M → A | < 70 Å^3^ | < 70 Å^3^ | 91.6 Å^3^ | 93.7 Å^3^ |
| IC_50_ AS_G | 0.49 µM ** | 6.64 µM | 0.63 ** | --- |
| IC_50_ AS_A | 4.22 µM * | --- | 6.21 * | --- |
| ipTM WT with ATP | 0.90 | 0.93 | 0.85 | 0.91 |
| ipTM AS_G with ATP | 0.90 | 0.92 | 0.80 | 0.92 |
| ipTM AS_A with ATP | 0.91 | 0.94 | 0.93 | 0.93 |
| **Table - Metrics from the predictive structural analysis of analog-sensitive variants of KKT2, KKT3, CRK9, and CLK1.**  IC_50_ measured in the *Leishmania* promastigote stage. Statistical significance compared with parasites expressing the wild-type kinase is indicated by asterisks (*, p-value < 0.05; ** p-value <0.01).  G, glycine; A, alanine; ipTM, interface predicted Template Modelling | | | | |

3- The resazurin assay reports metabolic activity rather than cell viability, so it cannot discriminate between dead cells and cells that are only growth-arrested. Therefore, statement in line 277 should be revised. To directly evaluate viability, the authors should complement the resazurin readout with a membrane integrity assay, such as propidium iodide staining, which distinguishes live from nonviable cells.

Thank you for pointing this out. We have corrected the text to provide a more precise description of the resazurin assay.

4- The IC50 of the parental strain is quite low, 11.42 uM in promastigote and 19.63 uM in intracellular amastigote (Figure 3), in the absence of naturally sensitive Leishmania kinases, the IC50 should be higher (above 100), as no targets are available. The authors should provide a potential explanation for these findings?

Our gatekeeper residue analysis identified 16 protein kinases that do not contain a bulky amino acid at the gatekeeper position: LmxM.36.4250 possesses a serine gatekeeper, whereas LmxM.15.1550, LmxM.05.0130, LmxM.10.0200, LmxM.10.0490, LmxM.13.1640, LmxM.14.1070, LmxM.21.1650, LmxM.29.0370, LmxM.29.2910, LmxM.02.0360, LmxM.21.0823, LmxM.28.0620, LmxM.21.0853, LmxM.31.1810, and LmxM.07.0880 harbour a threonine gatekeeper. Gatekeeper residues such as serine or threonine have been reported to influence the selectivity profile of bumped kinase inhibitors (BKIs). Among these kinases, LmxM.36.4250, LmxM.13.1640, LmxM.21.1650, LmxM.29.0370, and LmxM.29.2910 have been pinpointed as required for amastigote in *in vivo* mouse footpad model. Although none of the kinases with serine or threonine gatekeepers were identified as required for promastigotes or amastigotes in *in vitro* macrophage infections, it remains possible that concurrent inhibition of several of these kinases contributes to the IC_50_ values observed in wild-type parasites across both models.

Despite this, the differences in IC_50_ values between the wild-type and the analog-sensitive mutants are sufficiently pronounced to support our conclusions. Additionally, each analog-sensitive line exhibits a distinct phenotype, including the phenotype recently reported for another kinase we are studying (mitotic spindle kinase, MSK) on BioRxiv (doi: https://doi.org/10.1101/2025.09.19.677349), reinforcing the specificity of our system.

We have updated the Discussion section to clarify that serine and threonine gatekeeper residues may modulate BKI selectivity, which could explain the relatively low IC_50_ values observed in wild-type parasites.

5- The authors need to clarify that the values reported lines 287 to 293 are extracted from reference 43, and not derived from Figure 3. This distinction should be made explicit in the text, as presently written, this paragraph is unclear and may mislead readers. In addition, these values should be presented alongside the values obtained from the amastigote results (this study) so that the reader can readily compare the two data sets.

We have revised the text to address the reviewer’s comments and to clarify that the data were extracted from the reference mentioned.

However, we have not adopted the suggestion to present these values alongside the amastigote results. The manuscript already presents the promastigote and amastigote data in separate paragraphs, and we prefer to maintain this structure as it provides clarity and readability.

6- It should be indicated what data or references underpin the following claim: “possible due to reduced stability of 1NA-PP1 within the acidic environment of the macrophage parasitophorous vacuole” (Line 317-318).

As this is a speculative hypothesis, we have removed the claim from the manuscript.

7- The authors are encouraged to replace Figure 4a with Supplemental Figure 11a, as the latter presents the data more clearly and is therefore more informative.

We accepted the suggestion and have updated the figures and their legends accordingly.

8- MNC: does this refer to multinucleated cells? The abbreviation is not defined anywhere in the text.

Thank you for pointing this out. We have defined MNC, consistent with the other abbreviations used for cell cycle phases, in the Result section, in the legends of Figures 6 and 7 as well as in the Supplementary Figures of the revised manuscript.

9- A clear rationale should be provided for the experimental conditions employed: (i) why a concentration of 5 µM was selected for the assay and why the specific time points of 6 h and 24 h were chosen; and (ii) why, in the parallel experiment using tagged KKT2, a higher concentration of 10 µM was used. Clarifying these decisions will help readers assess the relevance and comparability of the results.

We have added an explanation of the rationale for the experimental conditions used to interrogate the analog-sensitive variants of KKT2 and CRK9 at the end of the Results section entitled “**KKT2 and CRK9 protein kinase activity is essential for *Leishmania*.**” The conditions were selected based on the parasite cell cycle length and the susceptibility of parasites expressing the analog-sensitive variants to 1NM-PP1. To support this rationale and to address the reviewer’s question whether inhibition of KKT2 by 1NM-PP1 induces parasite cell death or instead affects cell proliferation (minor point 12, reviewer 3), we have added panel “d” and “e” to Fig. 4 of the revised manuscript.

10- In Figures 4 and Supplementary Figure 11, the data should be interpreted relative to the treated wild type control rather than to the untreated wild type. At the 6 hour time point (Supplementary Figure 11a) the treated wild type exhibits a reduction of the G₁ population and a concomitant increase in S phase cells, changes that are not observed in the untreated wild type. This shift reveals that 1NM-PP1 itself affects cell cycle progression in the wild type strain, which can alter the interpretation of the KKT2 AS data. For example, at the 24 hour time point, the increase in S phase cells observed in the KKT2 AS is also present in the treated wild type control and is therefore not a phenotype of KKT2 inhibition as stated lines 344 345. Therefore, all subsequent analyses should be compared to the treated wild type condition rather than to the untreated control.

As recommended by the reviewer, we performed statistical comparisons between the wild-type and analog-sensitive lines under treated conditions, and we have incorporated these results into the corresponding figures. We have also retained the original comparison between treated and untreated AS lines, as minor differences in culture timing between the two cell lines may occur. The manuscript has been revised accordingly.

11- Figure 4d would benefit from additional microscopy data to substantiate the reported effects on the cell cycle progression of the mutant: (i) images representing cell cycle stages 1 and 5 (also absent from Figure 13) might be included to provide a complete view of the phenotypes observed in the mutant population. Second, corresponding images of wild type cells treated with 1NM PP1 is necessary, because the inhibitor itself impacts the cell cycle; this control will allow direct comparison between treated wild type and mutant cells. Finally, DIC images should be added for each condition so that flagellum duplication can be clearly visualized. Including these elements will give readers a comprehensive and balanced representation of the cell cycle alterations.

Unfortunately, for the Elyra7 super-resolution microscopy experiments under treated conditions, we focused exclusively on capturing images of the inhibited phenotype. As a result, we do not have images corresponding to stages 1 – 5 for these conditions on KKT2 AS. The parental T7/Cas9 control was used in our widefield fluorescence imaging experiments to assess the effect of KKT2 inhibition on *Leishmania* cell cycle distribution (Fig. 6b). Data for both untreated and treated T7/Cas9 parasites were included in the revised manuscript Fig bb.

Regarding DIC imaging, we are unable to acquire DIC images using the Elyra because the DIC optical components interfere with the Lattice SIM. Alternatively, we used flagellar positioning with the widefield fluorescence microscope (image analysis presented in Fig 6b).

12- The percentage of cell death at the two time points, 6h and 24h should be provided, to determine the fate of the arrested cells in KKT2-AS treated samples, whether they recover or die.

We assessed cell death and proliferation for the glycine analog-sensitive variants of KKT2, CRK9 as well as the parental T7/Cas9 control. Inhibition of CRK9 and KKT2 initially resulted in a significant reduction in cell proliferation after 6 and 24 h, respectively, whereas loss of viability was observed only after 48 h of inhibition, indicating a primarily cytostatic effect and that sustained inhibition ultimately leads to parasite death. Consistent with a time-dependent effect, the majority of cells had lost viability after one week of treatment. In the KKT2 AS line, 51.3% and 63.2% of cells were non-viable following treatment with 5 µM and 10 µM 1NM-PP1, respectively, whereas in the CRK9 AS line the corresponding proportions of non-viable cells were 84.3% and 89%. Importantly, these conditions did not impair growth or viability of the parental T7/Cas9 control line (Fig 4d). To determine whether cells arrested upon KKT2 AS inhibition were capable of recovery, we performed washout experiments in which the inhibitor was removed after 6 or 24 h of treatment, and clone recovery was quantified relative to untreated controls. Even after 6 h of inhibition, the proportion of recovered clones was significantly reduced. Following 24 h of treatment, only 1.05% of clones recovered after exposure to 5 µM 1NM-PP1, and no recovery was observed at 10 µM (Fig. 4e). These findings demonstrate that KKT2 inhibition rapidly compromises parasite proliferative capacity and that even short-term inhibition markedly reduces the ability of cells to recover, with prolonged inhibition resulting in an essentially irreversible loss of proliferative potential.

These results have been added as panel “d” and “e” of Fig 4 in the revised manuscript.

13- The authors should evaluate whether the mNG-tag fused to KKT2 AS alters parasite growth, viability, and cell cycle progression. This assessment should be performed by comparing the KKT2 AS mNG strain with a control line expressing unmodified KKT2 mNG, both in the presence and in the absence of 1NM PP1.

To assess whether fusion of the mNG tag to KKT2 or KKT2_AS affects parasite growth and, consequently, cell-cycle progression, we compared cumulative growth of the KKT2_AS::mNG::3xMyc, KKT2::mNG::3xMyc, KKT2_AS and T7Cas9 cell lines. No significant differences were observed, and these data are now presented in Supplementary Fig 17 of the revised manuscript. Importantly, the KKT2_AS::mNG::3xMyc line enabled visualization of all parasite cell-cycle stages by super-resolution microscopy. Furthermore, because KKT2 is an essential protein, any functional disruption caused by the mNG tag would likely prevent recovery of a cell line in which both endogenous alleles are tagged. Therefore, the successful tagging of both alleles provides a practical indication that the mNG tag does not impair essential KKT2 function.

14- In Supplementary Figure 14, the 24 hour time point shows a clear rise in MNC together with a concomitant reduction of cells in S phase; however, these changes are not addressed in the text. The authors should discuss these observations and explain their relevance, if any, to the experimental outcomes.

As recommended by the reviewer, we have addressed these changes in the revised manuscript.

15- A clear schematic that illustrates the DMSO versus SM1‑71‑biotin experiment and the subsequent competitive chemoproteomics workflow should be provided. A visual representation of the labelling, enrichment, competition steps, and mass‑spectrometry analysis would greatly aid reader comprehension, as the current description and the Materials‑and‑Methods section are insufficiently detailed.

A schematic illustrating the SM1-71 experimental workflow has been added to Fig. 8a in the revised manuscript. This emphasises that in the competition experiment, any proteins which bind covalently to SM1-71 during pre-incubation will have their binding-sites permanently blocked for interaction with SM1-71-biotin and so will be under-represented under these pull-down conditions.

We have included more details of the protocol in the methods

16- In the competition experiment (lines 460 478), 4 µM SM1 71 was used, but, unless I missed it, it does not indicate the concentration of SM1 71 biotin that was added subsequently. The assay is performed on cell lysates, suggesting the absence of washing steps that can remove the SM1 71 and the SM1 71 biotin before the overnight incubation with streptavidin beads. Consequently, competition between the two compounds continues during the pull down, can it affect purification? It is also unclear whether the amount of streptavidin resin used in the protocol is sufficient to capture all PK-bound SM1 71 biotin, considering that the beads could become saturated with free SM1 71 biotin. This could affect enrichment efficiency. The authors should comment on those points.

We have revised the material and methods section “SM1-71 affinity purification” to include the concentration of SM1-71-biotin used (4 µM). Also included are more details for the streptavidin-linked beads (product code and protein binding capacity). As the new schematic (Fig 8a) shows, a decreased representation of any proteins suggests that their binding sites are blocked during pre-incubation.

It is unlikely that the beads (1mg, protein binding capacity ≥ 0.3 mg/mL IgG) are saturated with SM1-71-biotin as naturally biotinylated lysate proteins are also pulled down during the process. Even if SM1-71-biotin should become bound to bead before being able to interact with an SM1-71-binding protein, the SM1-71 moiety and warhead should still be available for interaction to occur with lysate proteins.

17- The order of the supplementary files is incorrect, Supplementary Data 3 appears before Supplementary Data 2.

Thank you for pointing this out. We have corrected the order of the supplementary files in the revised manuscript.

**Reference:**

1.	Baker N, Catta-Preta CMC, Neish R, Sadlova J, Powell B, Alves-Ferreira EVC, et al. Systematic functional analysis of Leishmania protein kinases identifies regulators of differentiation or survival. Nat Commun. 2021;12(1):1244. Epub 2021/02/25. doi: 10.1038/s41467-021-21360-8. PubMed PMID: 33623024; PubMed Central PMCID: PMCPMC7902614.
